# Improved model predictive current control strategy for overvoltage suppression in long-cable PWM-driven induction motor electrical submersible pump systems

**DOI:** 10.1371/journal.pone.0360296

**Published:** 2026-10-29

**Authors:** Xiaopeng Gong, Xin Qi, Ming Feng, Liancheng Zhu, Nianxing Wang

**Affiliations:** 1 School of Mechanical Engineering, University of Science and Technology Beijing, Beijing, China; 2 School of Electrical Engineering, Liaoning University of Technology, Jinzhou, China; 3 Daqing Oilfield Powerlift Industrial Pump Co. Ltd, Daqing, China; Qingdao University, CHINA

## Abstract

Long-cable PWM-driven induction motor electric submersible pump (ESP) systems suffer from significant voltage reflection issues caused by distributed parameter effects, leading to transient overvoltages at the motor terminals that can reach several times the DC bus voltage. This severely threatens system stability and motor insulation integrity, and traditional control methods prove inadequate for effective suppression. To address this challenge, the paper proposes an improved model predictive current control (MPCC) strategy for induction motor ESP systems, which integrates overvoltage suppression and equivalent switching frequency reduction for long-cable PWM-driven applications. The reflection overvoltage mechanism is thoroughly analyzed through mathematical modeling of the motor and long-cable transmission line, and the applicability of predictive current control in such systems is investigated. The core improvement lies in a circular boundary constrained MPCC combined with a dynamic transition voltage vector selection mechanism, which enables dual-mode optimization based on current error boundary conditions: the normal cost function is applied within the circular boundary to jointly optimize voltage tracking, switching frequency, and dv/dt; while the backup cost function is activated outside the boundary to prioritize current error minimization. This effectively optimizes the voltage switching strategy, reducing both switching frequency and dv/dt, thereby preventing overvoltage excitation caused by abrupt voltage vector steps in PWM-driven systems. Simulation models under different cable configurations (1 km with RLC filter and 110 m without RLC filter) and an experimental platform based on a neutral-point-clamped three-level inverter (NPC-TLI) topology are established, with the 110 m no-filter configuration serving as a direct cross-validation baseline. Comparative simulation and experimental results demonstrate that the proposed strategy significantly reduces the duration of motor terminal overvoltage while maintaining excellent system dynamic performance. Moreover, the improved MPCC effectively suppresses overvoltage and reduces equivalent switching frequency without requiring hardware filter redesign, while the RLC filter remains necessary for full insulation safety compliance under more demanding long-cable conditions, substantially mitigating the risk of motor insulation breakdown.

## Introduction

Electrical submersible pump (ESP) systems powered by long-cable pulse-width modulation (PWM)-driven induction motors represent critical infrastructure in contemporary energy extraction operations, being widely deployed across petroleum, geothermal, and deep-water applications where well depths routinely exceed 1 km [1]. The operational environment of these systems presents extraordinary challenges: down-hole temperatures surpassing 150 °C, corrosive fluid mixtures, and power transmission distances extending up to 3–5 km between surface-mounted inverters and submersed motors [2,3].

Recent studies have extensively investigated various failure mechanisms in electrical submersible pump (ESP) systems. Among these, voltage reflection-induced transient overvoltage has been identified as a critical factor affecting motor insulation integrity, which can drastically reduce motor lifespan in severe cases [4–6]. These overvoltage spikes severely stress the winding insulation, leading to partial discharges, accelerated aging, and eventual motor breakdown [7]. The economic impact is substantial, as maintenance interventions in such environments incur extremely high costs related to rig mobilization and deferred oil production [8,9].

Recent advances in power electronic system modeling have also contributed to improved understanding and mitigation of such transients. A derivative-free grid inertia estimation method for converter-dominated power systems has been proposed, demonstrating the broader trend toward robust and computationally efficient control strategies in modern power electronics applications [10].

This vulnerability originates fundamentally from distributed parameter effects in lengthy power cables. At typical PWM switching frequencies (2–20 kHz), cables beyond 500 m exhibit transmission line behavior characterized by surge impedance $Z_{c}=\sqrt{L_{0}/C_{0}}$ [11]. When this impedance mismatches the motor’s significantly lower surge impedance ($Z_{m}<50\;\Omega$), voltage reflection occurs, generating transient overvoltages at the motor terminals that can reach 2–3 times DC bus voltage or more [12,13]. Various filter networks have been proposed to mitigate these overvoltages and their influence on motor voltage distribution and common-mode currents [14,15].

Accurate modeling of the long cable is essential for understanding the overvoltage propagation mechanism. A transmission line modeling method based on the vector fitting algorithm has been proposed [16], which extracts a rational approximation function from frequency response analysis data to construct an equivalent RLC network for physical implementation. This approach provides a systematic framework for representing the frequency-dependent characteristics of transmission lines, which is particularly relevant to the filter parameter challenges discussed later in this work.

Compounding the problem, insulation degradation follows inverse power-law kinetics where lifetime $L=k\cdot V^{-n}$ with exponent n≈12 for modern enameled wires—meaning a moderate overvoltage increase can reduce motor lifespan by over 90% [17]. This relationship has been widely validated in the literature: a larger voltage endurance coefficient indicates better insulation endurance, while excessive overvoltage dramatically shortens motor service life [18]. Given the high cost of interventions in remote offshore or deep-well installations, developing robust overvoltage suppression strategies becomes not merely a technical challenge but an economic imperative.

Conventional mitigation strategies remain constrained by fundamental physical and economic trade-offs that limit their applicability in field conditions. Passive hardware solutions, while mechanically robust, introduce significant performance compromises. Optimized resistor-capacitor (RC) snubber circuits reduce overvoltage amplitude but incur substantial power losses at rated load, increasing operational costs for typical ESP systems [12,14]. However, common-mode chokes are recognized as key components that contribute significantly to the weight and volume of a drive system, particularly in applications with stringent power density requirements [19,20]. Ferrite beads, despite their compact size, exhibit nonlinear permeability characteristics that can distort PWM waveforms [21]. This distortion induces additional bearing currents, which may accelerate mechanical wear in the motor. Active damping techniques, which modulate inverter output impedance, offer theoretical adaptability. However, their performance is critically dependent on the real-time, precise identification of cable parameters; estimation errors can substantially increase overvoltage magnitude, and passive filtering solutions such as *du*/*dt* filters incur additional power losses that scale with filter parameters and cable length [22].

The selection of PWM modulation techniques fundamentally influences the trade-off between inverter efficiency and motor current quality. A comprehensive simulation study comparing eight PWM techniques for permanent-magnet synchronous motor drives reveals that space vector modulation (SVM) achieves the lowest current THD (approximately 5.0%), while 60° discontinuous PWM (DPWM) yields the highest inverter efficiency [23]. These findings underscore the inherent conflict between harmonic suppression and switching loss reduction, which motivates the development of advanced control strategies that can achieve a more balanced performance trade-off.

Seeking to overcome the limitations of hardware-based solutions, research has increasingly focused on software-based control strategies. Among these, finite-control-set model predictive control (FCS-MPC) has emerged as a prominent candidate due to its fast dynamic response and inherent ability to handle system constraints. Traditional FCS-MPC for motor drives typically formulates a cost function aimed at minimizing future current tracking error. While this approach performs well for torque and flux control, it exhibits a critical flaw in long-cable applications: the controller is fundamentally agnostic to wave propagation dynamics within the cable. As demonstrated in recent studies, the characteristic impedance mismatch between transmission line cables and motor coils induces reflected wave over-voltage (RW-OV) at the motor terminals [24]. Moreover, under low-speed operating conditions where narrow PWM pulses are frequent, a more severe double-pulsing phenomenon can occur, where a new switching event takes place before the oscillation from the previous switching has fully decayed, resulting in overvoltage oscillations exceeding 3 p.u. [24].

The controller’s optimization relies on a simplified plant model—often the motor alone—disregarding transmission line behavior. In pursuing optimal current tracking, the algorithm frequently selects voltage vectors causing abrupt output changes. Various PWM-based techniques have been proposed to mitigate motor overvoltage, but conventional methods such as pulse elimination and maximum minimum pulse technique distort the voltage waveform and degrade output current characteristics, potentially leading to control instability [25]. Hardware-based solutions combining RLC filters with PWM techniques have been developed, where the filter damping resistance is matched to cable impedance to attenuate reflected overvoltage [12]. Thus, a control method prized for dynamic performance can paradoxically aggravate the very problem it seeks to solve, creating a vicious cycle where improved dynamic response accelerates insulation degradation. This issue is compounded in sensorless drives, where voltage distortion from reflections corrupts back-EMF estimation, leading to speed and position errors that degrade control stability [26].

Bridging the research gap requires a control paradigm that combines the adaptability of model-based predictive control with explicit overvoltage suppression. Several technical challenges remain. First, integrating a full distributed-parameter cable model like the Bergeron model into real-time MPC is computationally prohibitive; while fast, the Bergeron model tends to overestimate overvoltage transients [27]. Second, the cost function must be re-engineered to jointly optimize dynamic performance and limit motor-terminal overvoltage, penalizing voltage trajectories that excite cable resonance. Third, voltage vector selection must avoid high-*du*/*dt* switches, especially in multi-level inverter topologies [28,29]. Finally, the interplay between refined control law and practical issues like parameter uncertainty and sensorless operation must be addressed.

This paper proposes an improved MPCC strategy with overvoltage suppression for neutral-point-clamped three-level inverter (NPC-TLI) induction motor (IM) ESP drives. First, the mathematical modeling of the ESP and long-cable PWM-driven system are established. Second, the overvoltage reflection mechanism in long-cable PWM-driven IM ESP system is analyzed. Third, an overvoltage-suppressed MPCC strategy based on a current circular trajectory and time-assist information is proposed, a low switching frequency voltage space vector selection and optimization criterion with neutral-point voltage balancing control for the NPC-TLI is also presented. Finally, a long-cable PWM-driven IM ESP control system incorporating the improved MPCC with or without an RLC filter is constructed. Comparative simulation and experimental analyses are then conducted against the traditional MPCC and SVPWM strategies. The results demonstrate the effectiveness and feasibility of the improved MPCC strategy.

## Mathematical modeling of IM ESP and long-cable PWM-driven system

### Mathematical modeling of IM ESP

In the synchronous rotating coordinate system aligned with the rotor flux, the stator current vector $i_{s}$ and rotor flux vector $\psi_{r}$ of the IM ESP are selected as state variables, leading to the derivation of following equations as (1) and (2) [30]:


$$\begin{array}{c} \left\{ \begin{array}{c} \tau_{\sigma }^{{\;}^{\prime }}\frac{{di}_{s}}{dt}+i_{s}=-j\omega_{s}\tau_{\sigma }^{{\;}^{\prime }}i_{s}+\frac{K_{r}}{R_{\sigma }}(\frac{1}{\tau_{r}}-j\omega_{r})\psi_{r}+\frac{1}{R_{\sigma }}u_{s}\;\tau_{r}\frac{{d\psi }_{r}}{dt}+\psi_{r}=-j(\omega_{s}-\omega )\tau_{r}\psi_{r}+L_{m}i_{s} \end{array}\right. \end{array}$$ (1)

$$\begin{array}{c} \left\{ \begin{array}{c} \tau_{\sigma }^{{\;}^{\prime }}=\sigma L_{s}/R_{\sigma }\\ R_{\sigma }=R_{s}+K_{r}^{2}R_{r} \end{array}\right. \end{array}$$ (2)

where the subscripts *s* and *r* stand for stator and rotor parameters, including resistance ($R_{s,r}$), inductance ($L_{s,r}$), flux linkage ($\psi_{s,r}$), current ($i_{s,r}$), and angular frequency ($\omega_{s,r}$), respectively; $\sigma =1-K_{s}K_{r}$ denotes the leakage inductance coefficient; $\omega_{s}$ and $\omega_{r}$ express stator and rotor electrical angular velocity; $K_{s}=L_{m}/L_{s}$, $K_{r}=L_{m}/L_{r}$, $L_{s}$, $L_{r}$ and $L_{m}$ represent the magnetic field coupling coefficient, self-inductance and mutual inductance coefficient, respectively; $R_{\sigma }$ is the equivalent resistance, $R_{s}$ and $R_{r}$ are the phase resistances of the stator and rotor; $\tau_{r}=L_{r}/R_{r}$ refers to the rotor time constant; $\tau_{\sigma }^{{\;}^{\prime }}$ is the transient stator time constant.

The electromagnetic torque $T_{e}$ of the IM can be given through the cross product of the rotor flux vector $\psi_{r}$ and stator current vector $i_{s}$ as (3):


$$\begin{array}{c} T_{e}=\frac{3}{2}p\frac{L_{m}}{L_{r}}(\psi_{r}\times i_{s}) \end{array}$$ (3)

where *p* is the number of pole-pairs, then the mechanical dynamic equation of the IM is expressed as (4):


$$\begin{array}{c} \tau_{m}\frac{d\omega_{rm}}{dt}+B\omega_{rm}=T_{e}-T_{L}=\frac{3}{2}p\frac{L_{m}}{L_{r}}(\psi_{r}\times i_{s})-T_{L} \end{array}$$ (4)

where $\tau_{m}$ is the mechanical time constant; $\omega_{rm}=2\pi n_{r}/60$ is rotor mechanical angular velocity; *B* is the viscous friction coefficient; $T_{L}$ is the load torque.

According to (1)-(4), one can obtain the signal flow diagram of the IM as presented in Fig 1.

**Fig 1:**
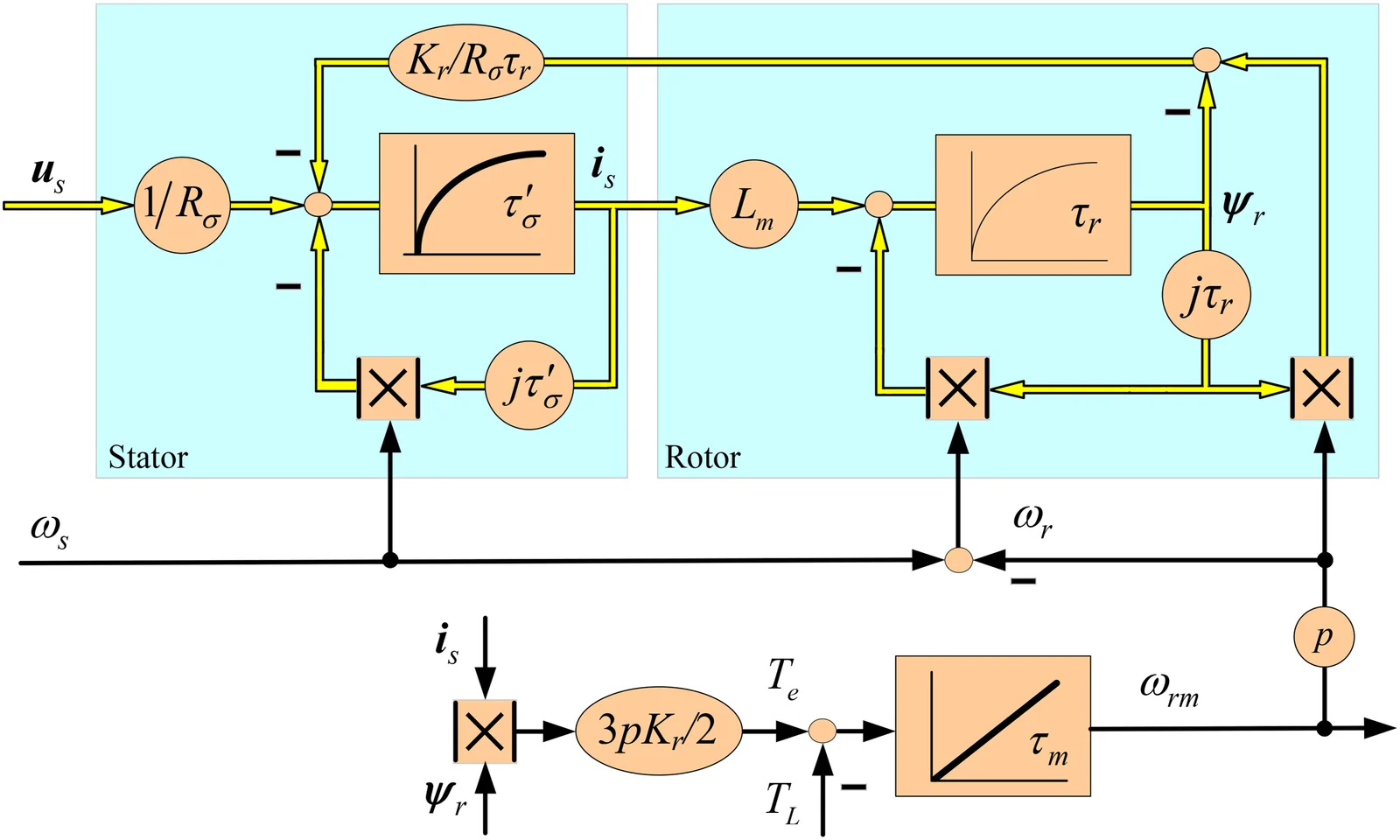
Induction motor signal flow diagram.

### Long-cable inverter PWM-driven system modeling based on transmission theory

To analyze and calculate the overvoltage impact on the IM ESP and provide a reliable basis for the subsequent design of special-purpose RLC filter parameters, the inverter-powered IM drive system with long-cable transmission lines and distributed parameter circuits is treated as an integrated circuit topology, which as illustrated in Fig 2.

**Fig 2:**
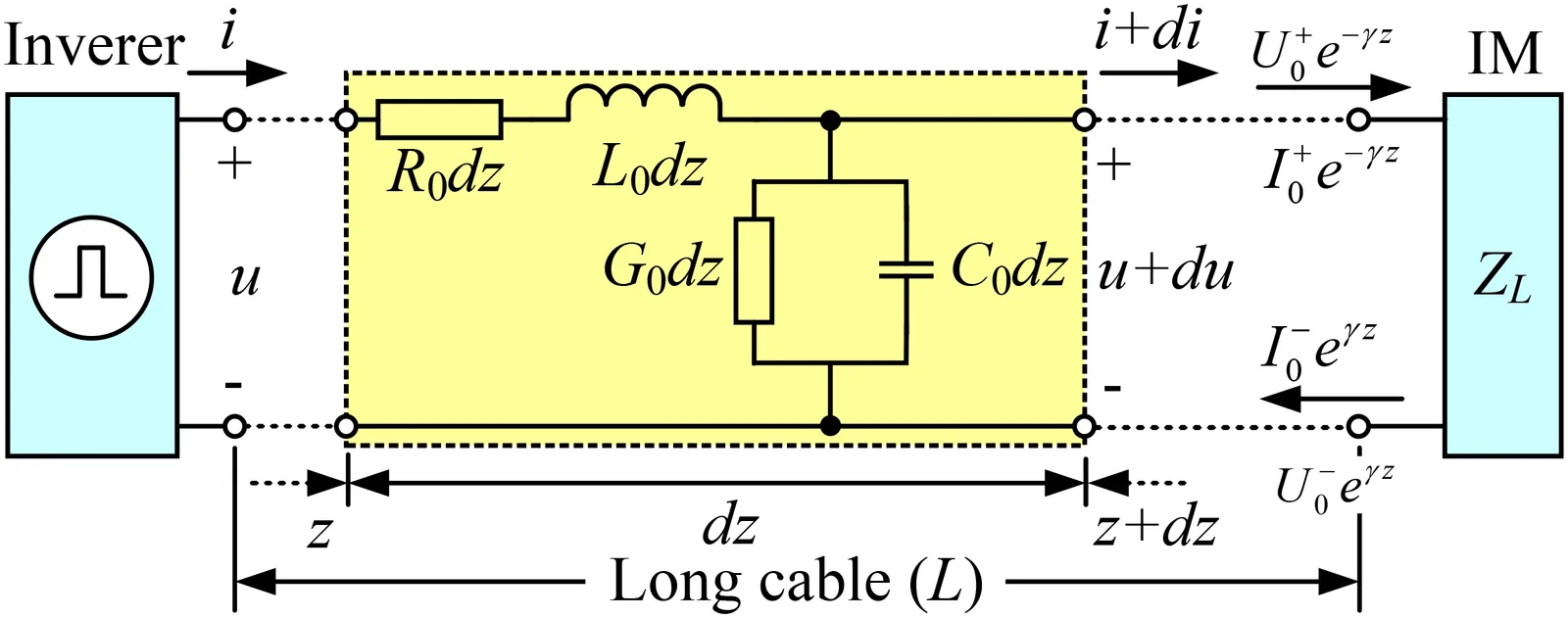
Inverter-long cable-induction motor ESP system topology.

As shown in Fig 2, where *u* is considered as the ideal PWM voltage source of the output from inverter; the long-cable transmission line (*L*) typically divided into numerous infinitesimal segments *dz*, each of which can be treated as a lumped parameter circuit; each infinitesimal segment consists of a Γ-shaped network comprising resistance *R*_0_, inductance *L*_0_, capacitance *C*_0_ and leakage conductance *G*_0_ per unit length (*dz*); $U_{0}^{+}$, $U_{0}^{-}$, $I_{0}^{+}$ and $I_{0}^{-}$ represent the amplitudes of forward and reverse traveling waves, respectively; $e^{-\gamma z}$ represents the forward wave propagating from the inverter to IM, decaying in amplitude and shifting in phase as *z* increases; $e^{\gamma z}$ denotes the reverse, reflected wave, caused by impedance mismatch between the IM and long-cable transmission line, which also decays and shifts in phase as it travels back toward the inverter; $Z_{L}$ is the equivalent impedance of the IM.

The high-frequency PWM signals (*u*) generated by the inverter cause long cable to exhibit notable transmission line effects. When the cable length is comparable to the wavelength of electromagnetic wave corresponding to the signal frequency, the distribution of voltage and current along the cable is no longer uniform but shows distinct traveling wave characteristics, resulting in variations in voltage and current values at different positions of the cable. In such cases, a transmission line model must be employed to accurately depict the electrical properties of the cable.

By applying Kirchhoff’s laws to Fig 2, the transmission line model, also known as the telegrapher’s equations, can be obtained as (5):


$$\begin{array}{c} \left\{ \begin{array}{c} \frac{\partial u(z,t)}{\partial z}=-R_{0}i(z,t)-L_{0}\frac{\partial i(z,t)}{\partial t}\\ \frac{\partial i(z,t)}{\partial z}=-G_{0}u(z,t)-C_{0}\frac{\partial u(z,t)}{\partial t} \end{array}\right. \end{array}$$ (5)

where, (5) can be represented using phasors as (6):


$$\begin{array}{c} \left\{ \begin{array}{c} u(z,t)=\text{Re}\left[U(z)e^{j\omega_{r}t}\right]\\ i(z,t)=\text{Re}\left[I(z)e^{j\omega_{r}t}\right] \end{array}\right. \end{array}$$ (6)

Substituting (6) into (5) rearranging the terms yields as (7) and (8):


$$\begin{array}{c} \left\{ \begin{array}{c} \frac{d^{2}u(z,t)}{dz^{2}}+\gamma^{2}u(z,t)=0\\ \frac{d^{2}i(z,t)}{dz^{2}}+\gamma^{2}i(z,t)=0 \end{array}\right. \end{array}$$ (7)

$$\begin{array}{c} \gamma =\sqrt{Z_{0}Y_{0}}=\alpha +j\beta =\sqrt{\left(R_{0}+j\omega_{r}L_{0})+(G_{0}+j\omega_{r}C_{0}\right)} \end{array}$$ (8)

where, γ is the propagation constant of a lossy long-cable transmission line; α is the attenuation constant, which indicates the degree of signal intensity attenuation per unit length (*dz*) and reflects the impact of factors as long-cable material, structure, and operating conditions on signal transmission efficiency; β is the phase constant, representing the phase change per unit length (*dz*) and reflecting the propagation speed and wavelength of the signal.

The general solutions of (7) can be derived as (9):


$$\begin{array}{c} \left\{ \begin{array}{c} u(z,t)=u_{\text{i}}(z,t)+u_{\text{r}}(z,t)=U_{0}^{+}e^{-\gamma z}+U_{0}^{-}e^{\gamma z}\\ i(z,t)=i_{\text{i}}(z,t)+i_{\text{r}}(z,t)=I_{0}^{+}e^{-\gamma z}+I_{0}^{-}e^{\gamma z} \end{array}\right. \end{array}$$ (9)

where, $u_{\text{i}}(z,t),i_{\text{i}}(z,t)$, *u*_r_(*z*,*t*) and *i*_r_(*z*,*t*) represent the inciden*t* wave vol*t*age, current, reflected wave voltage and current, respectively, and the subscript letters i, u are represented in roman type.

From (9), it can be observed that the expressions for voltage and current consist of two components: one is the wave propagating along the -*z* axis, known as the incident wave; the other is wave propagating along the + *z* axis, referred to as the reflected wave.

Taking (8) and (9) into consideration, it can be concluded as (10).


$$\begin{array}{c} i(z,t)=\frac{\gamma }{R_{0}+j\omega_{r}L_{0}}(U_{0}^{+}e^{-\gamma z}-U_{0}^{-}e^{\gamma z}) \end{array}$$ (10)

Comparing (9) with (10), one can obtain:


$$\begin{array}{c} Z_{0}=\frac{U_{0}^{+}}{I_{0}^{+}}=\frac{U_{0}^{-}}{I_{0}^{-}}=\frac{R_{0}+j\omega_{r}L_{0}}{\gamma }=\sqrt{\frac{R_{0}+j\omega_{r}L_{0}}{G_{0}+j\omega_{r}C_{0}}} \end{array}$$ (11)

where, *Z*_0_ is the characteristic impedance of the transmission line, which defines the complex form of the ratio of voltage to current when the transmission line is infinitely long or has an extremely long electrical length; $Z_{0}=Z_{c}=\sqrt{L_{0}/C_{0}}$ with ignoring the influence of *R*_0_ and *G*_0_, i.e., the long cable is treated as a lossless transmission line (*R*_0_ = 0) with no dielectric losses occurring in its insulation (*G*_0_ = 0).

## Mechanism of overvoltage reflection and its corresponding RLC filter design

### Mechanism of overvoltage reflection for long-cable PWM-driven IM ESP system

When there is an impedance discontinuity in the long-cable PWM-driven IM ESP system, i.e., the impedance mismatch at the connection between long cable and motor, the signal will be reflected back in the opposite direction as a reflected wave, while the remaining part will continue to propagate along the transmission line as an incident wave with varying amplitudes. This generates destructive transient voltage spikes in the system.

The traveling wave reflection process of the long-cable PWM-driven IM system is illustrated in Fig 3. The reflected wave at a segment terminal end (*l* point) will propagate back along the transmission line to the starting end (0 point), where a portion of it will be reflected back to the terminal end again. This process repeats continuously, which will pose a risk of overvoltage at the terminal end, threatening the winding insulation of the IM ESP and reducing its service life.

**Fig 3:**
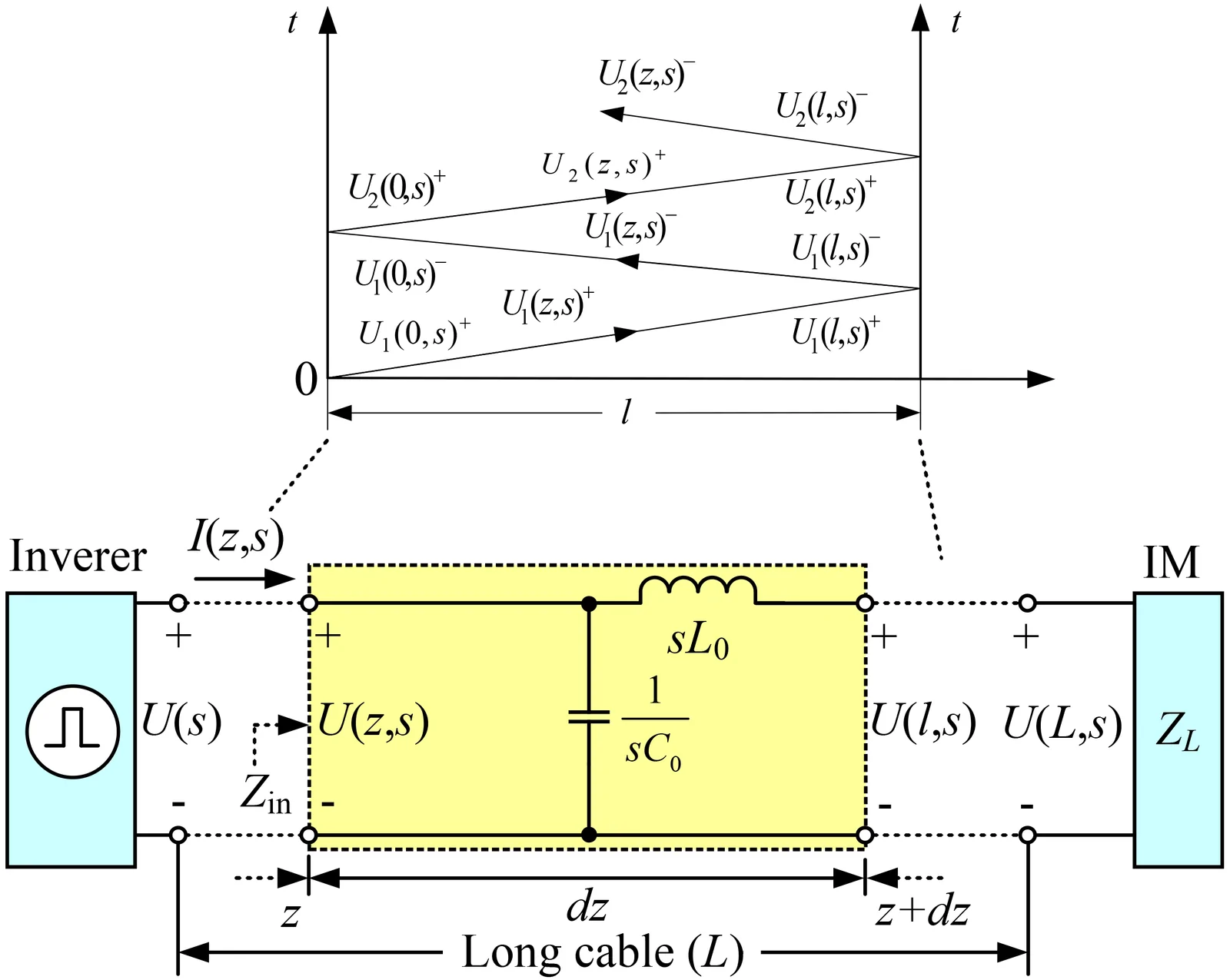
Mechanism of overvoltage reflection with long-cable PWM-driven IM ESP system.

As shown in Fig 3, assuming the long-cable transmission line connecting the inverter and IM is a lossless cable and with no dielectric losses occurring in its insulation, then its characteristic impedance is $Z_{0}=\sqrt{L_{0}/C_{0}}$; *s* is the Laplace operator; *l* represents the length of a segment of the *l*ong-cable transmission line, when referring to the entire distance from the inverter output end to the IM-side terminal end, *l* is equal to the tota*l* length *L* of the long cable; *U*(*z*,*s*) and *U*(*l*,*s*) denote the Laplace transform of the vo*l*tage at a specific point on the transmission line, measured at a distance of *z* and *l* from the inverter output end, respectively. Thus, at the *l*ong-cable starting end connecting to the inverter output, the voltage of *z* = 0 point can be expressed as *U*(*z*,*s*) = *U*(0,*s*) = *U*(*s*), while the IM-side terminal end voltage (*l* = *L*) is *U*(*L*,*s*).

Define the voltage reflection coefficient at location *z* as the ratio of the reflected wave voltage *u*_r_(*z*) to the incident wave voltage *u*_i_(*z*) at the same position, which can be expressed in (12).


$$\begin{array}{c} \Gamma_{z}=\frac{u_{\text{r}}(z,t)}{u_{\text{i}}(z,t)}=\frac{U_{0}^{-}e^{\gamma z}}{U_{0}^{+}e^{-\gamma z}} \end{array}$$ (12)

Substituting (12) into (9) yields (13).


$$\begin{array}{c} \left\{ \begin{array}{c} u(z,t)=u_{\text{i}}(z,t)\left[1+\Gamma_{z}\right]\\ i(z,t)=i_{\text{i}}(z,t)\left[1-\Gamma_{z}\right] \end{array}\right. \end{array}$$ (13)

Therefore, the input impedance at *z* point can be expressed as (14), then the $\Gamma_{z}$ can be obtained as (15).


$$\begin{array}{c} Z_{\text{in}}(z,t)=\frac{u_{\text{i}}(z,t)\left[1+\Gamma_{z}\right]}{i_{\text{i}}(z,t)\left[1-\Gamma_{z}\right]}=Z_{0}\frac{1+\Gamma_{z}}{1-\Gamma_{z}} \end{array}$$ (14)

$$\begin{array}{c} \Gamma_{z}=\frac{Z_{\text{in}}(z,t)-Z_{0}}{Z_{\text{in}}(z,t)+Z_{0}} \end{array}$$ (15)

Furthermore, one can derive the voltage reflection coefficients corresponding to the long-cable starting end (*z* = 0) and the IM-side terminal end (*z* = *L*), respectively, which are expressed in (16):


$$\begin{array}{c} \left\{ \begin{array}{c} \Gamma_{0}=\frac{Z_{\text{inv}}-Z_{0}}{Z_{\text{inv}}+Z_{0}}\\ \Gamma_{L}=\frac{Z_{L}-Z_{0}}{Z_{L}+Z_{0}} \end{array}\right. \end{array}$$ (16)

where *Z*_inv_ is the equivalent output impedance of the inverter (internal resistance, $R_{G}$). If $Z_{\text{inv}}=Z_{0}$, then $\Gamma_{0}=0$, meaning no reflected wave is generated and the impedances are perfectly matched; If $Z_{\text{inv}}\neq Z_{0}$, then $\Gamma_{0}\neq 0$, which will induce voltage reflection and further trigger overvoltage.

As shown in Fig 3, considering the sum expressions of (9) in the complex frequency domain *s* as *U*(*z*,*s*) and *I*(*z*,*s*), which can be concluded that during the propagation of the traveling wave along the long-cable transmission line, the voltage wave attenuates to its initial attenuation value from the starting end to the terminal end as $U_{1}(l,s{)}^{+}=$ $U(s)e^{-\gamma l}$. In addition, when the voltage wave reaches the terminal end, a portion of it, i.e., $U_{1}(l,s{)}^{-}=\Gamma_{L}U(s)e^{-\gamma l}$, will be reflected back to the starting end as $U_{1}(0,s{)}^{-}$, with the amplitude of the reflected voltage wave being $U_{1}(0,s{)}^{-}$ = $\Gamma_{L}\Gamma_{0}U(s)e^{-2\gamma l}$. After the reflected wave arrives at the starting end again, a portion of it will be reflected back to the terminal end once more, and the amplitude of the voltage wave generated by this reflection is $U_{2}(0,s{)}^{+}$ = $\Gamma_{L}\Gamma_{0}U(s)e^{-2\gamma l}$. This repeated reflection process generates a new voltage wave, forming an infinite series, which can be given as (17):


$$\begin{array}{cc} U(L,s)= & U(s)e^{-\gamma l}+U(s)e^{-\gamma l}(\Gamma_{L}\Gamma_{0})e^{-2\gamma l}+U(s)(\Gamma_{L}\Gamma_{0})e^{-2\gamma l}(\Gamma_{L}\Gamma_{0})e^{-3\gamma l}+...\\ = & \frac{U(s)e^{-\gamma l}}{1-\Gamma_{L}\Gamma_{0}e^{-2\gamma l}} \end{array}$$ (17)

where the denominator is less than 1 (because $\Gamma_{L}\Gamma_{0}$ is positive and less than 1), resulting in an amplitude of *U*(*L*,*s*) much greater than the output voltage of the inverter. When the cable length exceeds the critical value (usually <50 m), the peak voltage at the motor terminal can even reach twice the DC bus voltage of the inverter.

### The corresponding RLC filter design

The configuration of the RLC filter connected to the inverter output is illustrated in Fig 4. Where, $L_{f},C_{f},R_{f}$ denote eh RLC filter inductance, capacitance and damping resistance, *R*_G_ is the series resistance (including the internal resistance of the inverter).

**Fig 4:**
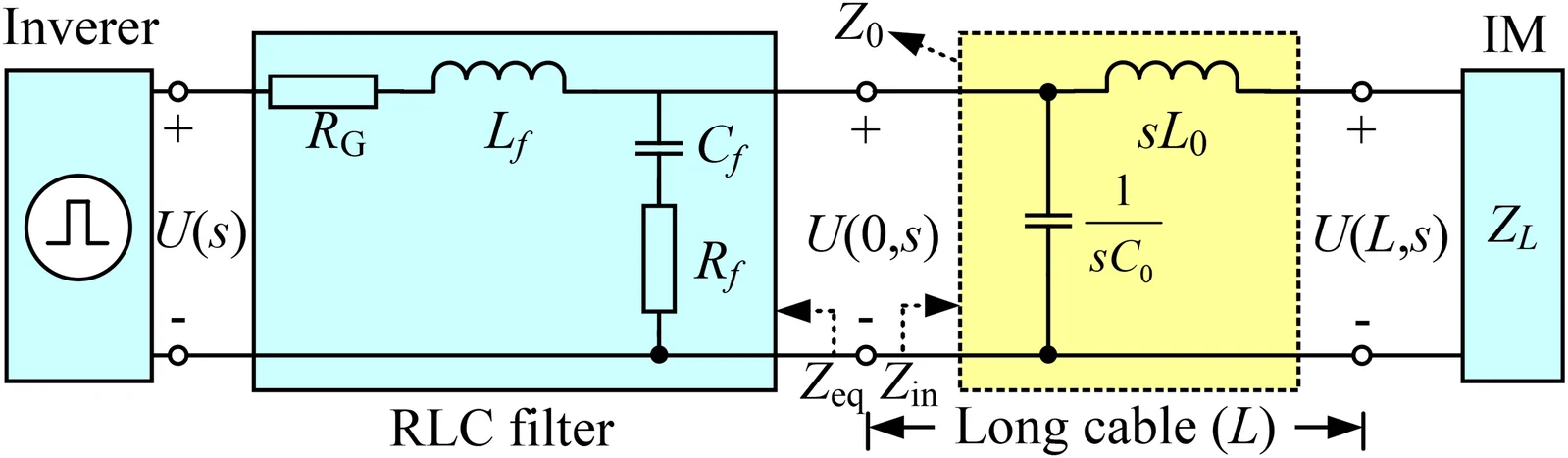
Topology of long-cable PWM-driven ESP system with RLC filter.

As shown in Fig 4, the RLC filter primary function is to reduce the voltage rate of change (*du*/*dt*) of the inverter PWM output voltage pulses. By extending the rise time of these pulses, the RLC filter can effectively mitigate overvoltage and high-frequency oscillations on the IM-side [16,24]. Based on Thevenin’s theorem, one can get the transfer function of RLC filter $G_{f}(s)$ as (18), allowing for $R_{\text{G}}\ll R_{f}$ under general operating conditions, i.e., $R_{\text{G}}\approx 0$, then $G_{f}(s)$ can be further simplified.


$$\begin{array}{c} G_{f}(s)=\frac{U(0,s)}{U(s)}=\frac{sR_{f}C_{f}+1}{s^{2}L_{f}C_{f}+sC_{f}(R_{\text{G}}+R_{f})+1}\approx \frac{sR_{f}C_{f}+1}{s^{2}L_{f}C_{f}+sC_{f}R_{f}+1}{|}_{R_{\text{G}}\approx 0} \end{array}$$ (18)

When the inverter and RLC filter are treated as a single active two-port network, its equivalent output impedance can be derived as (19):


$$\begin{array}{c} Z_{eq}(s)=\frac{\left(sR_{f}C_{f}+1\right)\left(R_{\text{G}}+sL_{f}\right)}{s^{2}L_{f}C_{f}+sC_{f}\left(R_{\text{G}}+R_{f}\right)+1}\approx \frac{\left(sR_{f}C_{f}+1\right)sL_{f}}{s^{2}L_{f}C_{f}+sC_{f}R_{f}+1}{|}_{R_{\text{G}}\approx 0} \end{array}$$ (19)

where the equivalent impedance accounts for the series-parallel combination of the filter’s resistance, inductance and capacitance, which is essential for analyzing the impedance matching between the RLC filter and the long-cable transmission line.

By comparing the simplified (18) with the canonical form of the transfer function for a standard zero‑point‑included second‑order system as (20), one can obtain (21):


$$\begin{array}{c} G(s)=\frac{2\zeta \omega_{n}s+\omega_{n}^{2}}{s^{2}+2\zeta \omega_{n}s+\omega_{n}^{2}} \end{array}$$ (20)

$$\begin{array}{c} \left\{ \begin{array}{c} \omega_{n}^{2}=\frac{1}{L_{f}C_{f}}\\ 2\zeta \omega_{n}=\frac{R_{f}}{L_{f}} \end{array}\right.\Rightarrow \left\{ \begin{array}{c} L_{f}=\frac{R_{f}}{2\zeta \omega_{n}}\\ C_{f}=\frac{1}{\omega_{n}^{2}L_{f}}=\frac{1}{2\zeta \omega_{n}R_{f}} \end{array}\right. \end{array}$$ (21)

where $\omega_{n}$ is the natural angular frequency (rad/s); ζ is the damping ratio; $R_{f}$ is generally set equal to the characteristic impedance *Z*_0_ for impedance matching.

## An Improved MPCC strategy for the long-cable PWM-driven IM ESP system

### Traditional current circular trajectory MPCC

The traditional block diagram of model predictive current control for PWM-driven IM system can be given as Fig 5 [31]. where the NPC-TLI topology and its PON voltage space vector position are illustrated in Fig 6 [32], and the basic idea of traditional current circular trajectory MPCC is to directly control the stator current of the IM to achieve fast current regulation.

**Fig 5:**
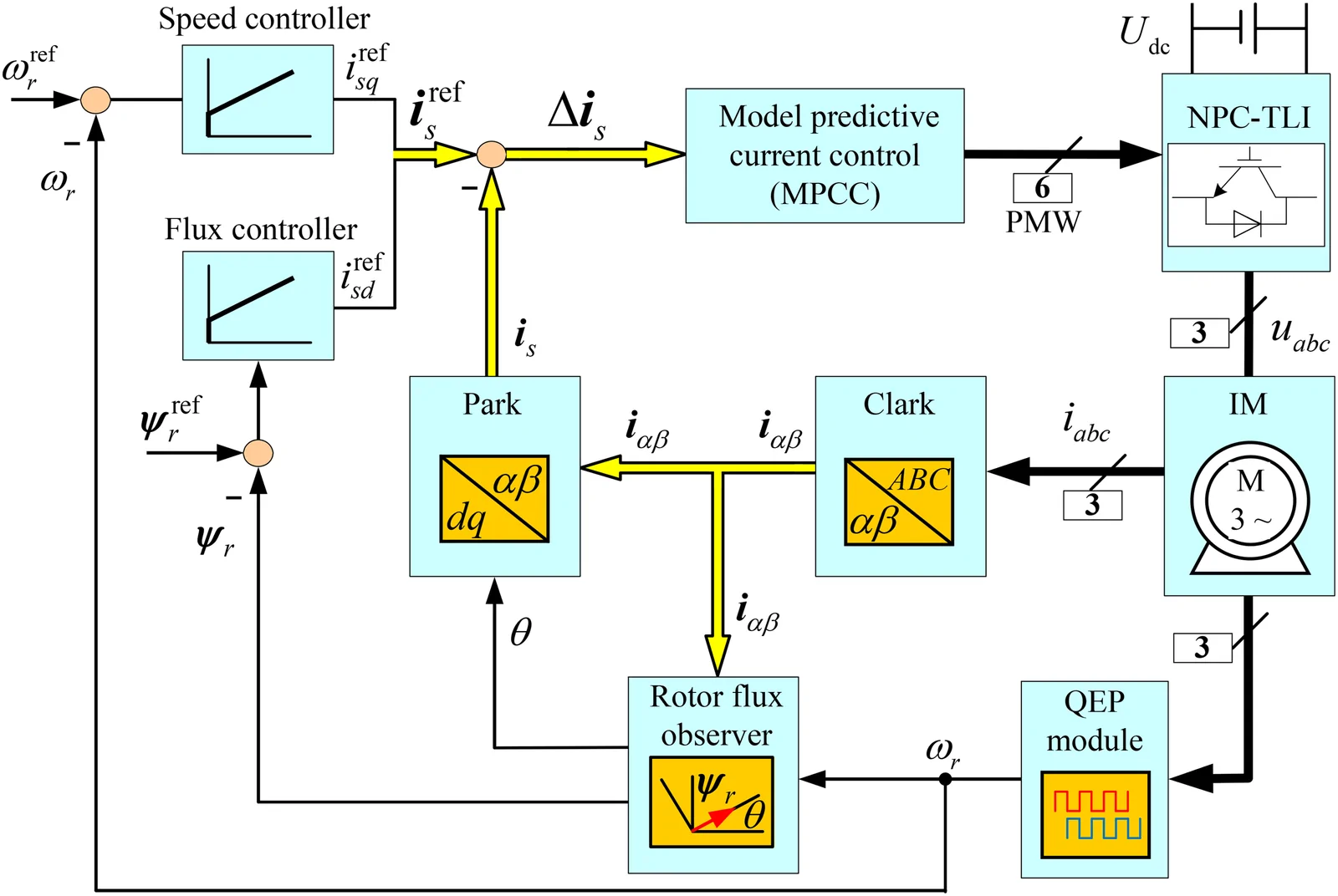
Block diagram of the traditional MPCC.

**Fig 6:**
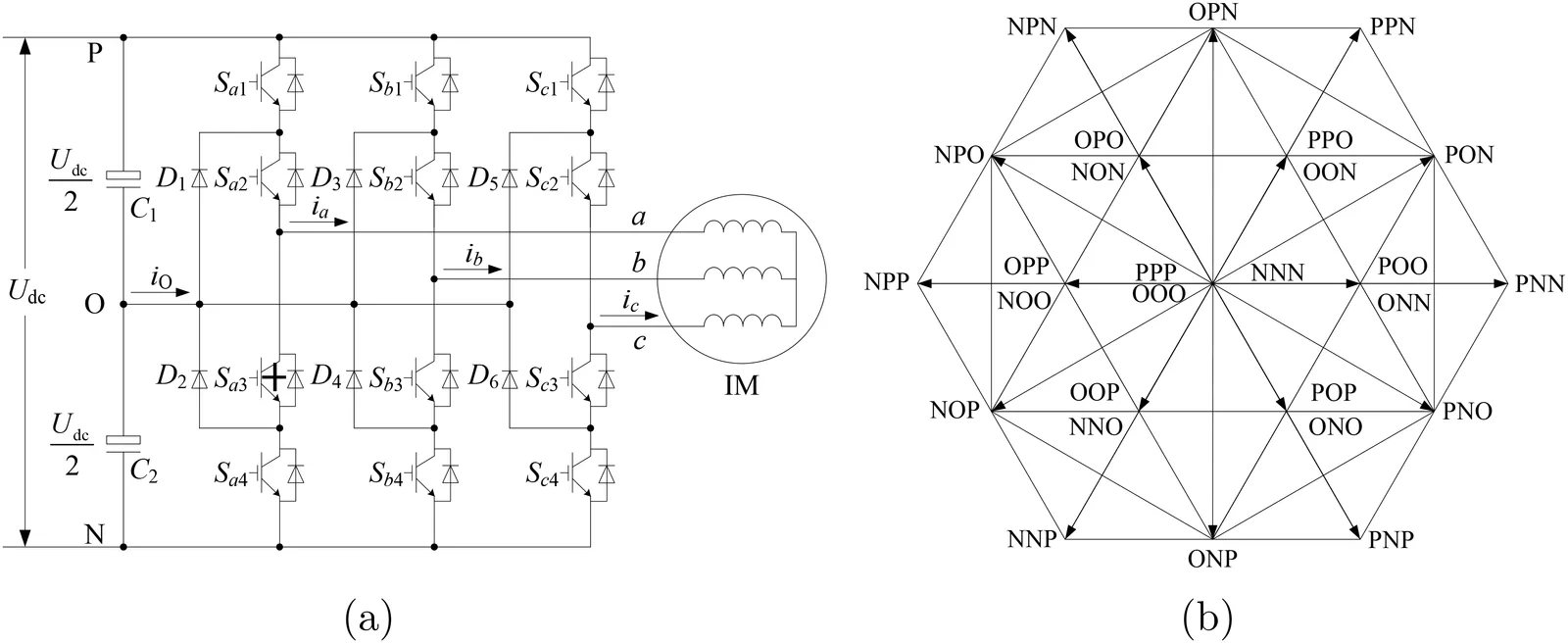
NPC-TLI topology and its PON voltage space vector positions. (a) Topology. (b) Voltage space vector position.

As shown in Fig 6(a), for a typical NPC-TLI, there are 3^3^  = 27 physical switching states corresponding to the possible P-O-N switching combinations. These 27 states map to 19 unique voltage vectors as shown in Fig 6(b), which can be classified into four categories in (22): one zero vector (3 switching states, all producing the same zero voltage), six small vectors (12 switching states, with two redundant states per vector), six medium vectors (6 switching states, each unique), and six large vectors (6 switching states, each unique).


$$\begin{array}{c} u_{sk}=\left\{ \begin{array}{cc} 0, & k=0\;(\text{Zero vector, 3 vectors})\\ \frac{1}{3}U_{dc}e^{j\frac{(k-1pi}{3}}, & k=1,2,...,6\;(\text{Small vectors, 12 vectors})\\ \frac{\sqrt{3}}{3}U_{dc}e^{j\left[\frac{(k-7)\pi }{3}+\frac{\pi }{6}\right]}, & k=7,8,...,12\;(\text{Medium vectors, 6 vector})\\ \frac{2}{3}U_{dc}e^{j\frac{(k-13)\pi }{3}}, & k=13,14,...,18\;(\text{Large vectors, 6 vectors}) \end{array}\right. \end{array}$$ (22)

where the zero, small, medium and large vectors totally are 27 voltage space vectors, which exactly match the 27 switching states of the NPC-TLI as shown in Fig 6(b). The indexed *k* directly corresponds to the lookup table entries, enabling efficient polling for optimal vector selection in MPCC.

Based on the NPC-TLI topology shown in Fig 6 and the voltage space vector expressions given in (22), the relationships among the switching states, the resulting neutral-point current, and the corresponding neutral-point voltage impact are summarized in Table 1.

**Table 1:** NPC-TLI switching states with neutral-point current and voltage impact.

*k*	Type	State	Neutral-Point Current *i*_O_	Neutral-Point Voltage Impact
0	Zero	PPP	0	No effect
		NNN	0	No effect
		OOO	$i_{a}+i_{b}+i_{c}$	Determined by currents
1	Small	POO / ONN	$-i_{a}$ / $i_{a}$	$i_{a}>0$: decreases / increases
2		PPO / OON	$-i_{c}$ / $i_{c}$	$i_{c}>0$: decreases / increases
3		OPO / NON	$-i_{b}$ / $i_{b}$	$i_{b}>0$: decreases / increases
4		OPP / NOO	$-i_{a}$ / $i_{a}$	$i_{a}>0$: decreases / increases
5		OOP / NNO	$-i_{c}$ / $i_{c}$	$i_{c}>0$: decreases / increases
6		POP / ONO	$-i_{b}$ / $i_{b}$	$i_{b}>0$: decreases / increases
7	Medium	PON	$i_{b}$	Same as $i_{b}$ (uncontrollable)
8		OPN	$i_{a}$	Same as $i_{a}$ (uncontrollable)
9		NPO	$i_{c}$	Same as $i_{c}$ (uncontrollable)
10		NOP	$i_{b}$	Same as $i_{b}$ (uncontrollable)
11		ONP	$i_{a}$	Same as $i_{a}$ (uncontrollable)
12		PNO	$i_{c}$	Same as $i_{c}$ (uncontrollable)
13	Large	PNN	0	No effect
14		PPN		
15		NPN		
16		NPP		
17		NNP		
18		PNP		

As illustrated in Table 1, among the four categories of voltage space vectors, only the small vectors possess two redundant switching states (P-type and N-type) that generate identical output voltages but produce opposite neutral-point currents. For example, the small vector corresponding to ONN and its redundant state POO produce the same voltage vector but draw current from or inject current into the neutral point in opposite directions. This unique property provides the necessary degree of freedom for active neutral-point voltage regulation. Medium vectors also affect the neutral-point voltage, but their neutral-point current direction is fixed and offers no flexibility for active balancing. Large vectors and zero vectors (except the OOO state) produce no neutral-point current and thus have no effect on the neutral-point voltage. Consequently, neutral-point voltage balancing can only be achieved when the candidate voltage vector belongs to the small-vector group, and this redundancy is exploited in the proposed control strategy to actively balance the neutral-point voltage without affecting the output voltage.

It is worth noting that the zero, small, medium and large vectors together constitute the 27 voltage space vectors, which exactly match the 27 switching states of the NPC-TLI shown in Fig 6(b). The indexed *k* directly corresponds to the lookup table entries, enabling efficient polling for optimal vector selection in the MPCC implementation.

As illustrated in Fig 7, a boundary circle is defined with the endpoint of the reference current vector $i_{s}^{\text{ref}}$ as the center and the allowable current error $\left|\Delta i_{s\text{max}}\right|$ as the radius. When the actual current vector $i_{s}$ lies inside the boundary circle, it indicates that the current distortion is within the allowable range $\left|\Delta i_{s\text{max}}\right|$, so there is no need to change the switching state of the inverter. When $i_{s}$ touches or exceeds the boundary circle, as indicated by the dashed line in Fig 7, it means that the current distortion is excessive, and the switching state of the inverter needs to be updated to adjust the voltage space vector $u_{sk}$ (*k* = 0–18 represents 19 out of the 27 voltage space vectors of the NPC-TLI in (22)) applied to the IM, thereby forcing the actual current vector $i_{s}$ to return to the interior of the boundary circle.

**Fig 7:**
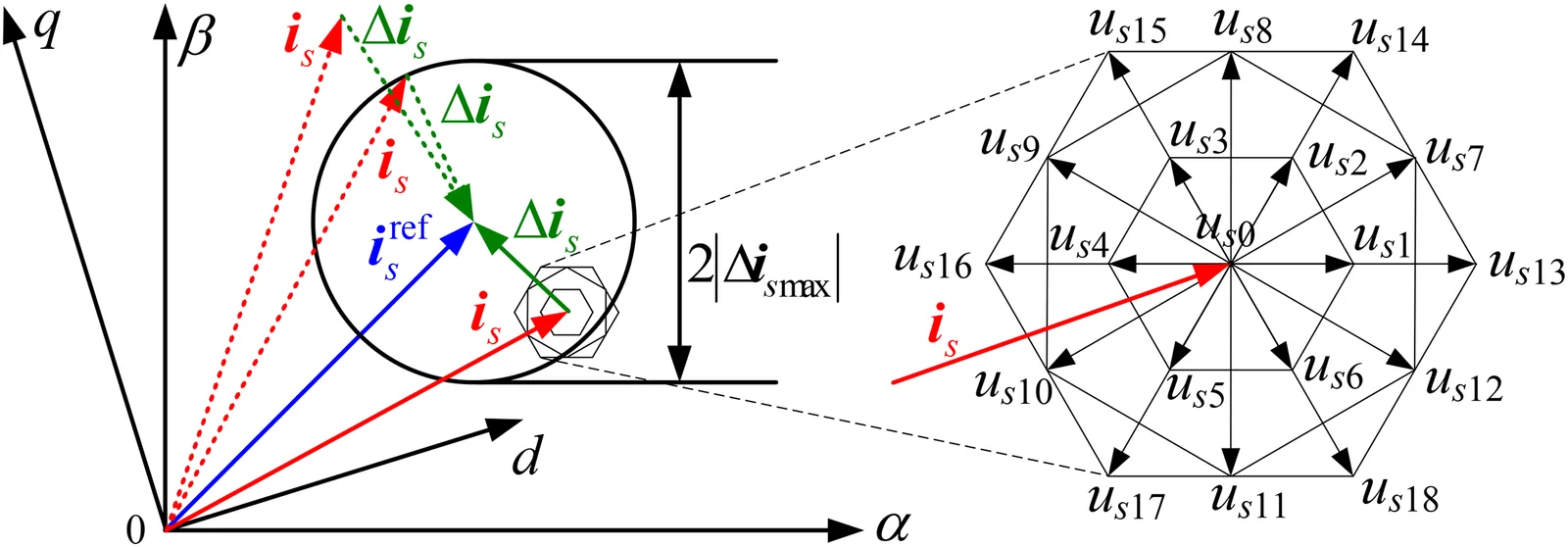
The principle of model predictive current control.

Here, one can summarize the implementation steps of the traditional MPCC as follows. First, sample the current, DC-link voltage, and neutral-point voltage, calculate $i_{s}$, $i_{s}^{\text{ref}}$, and $\Delta i_{s}$ at the current moment, and generate all combinations of driving signals for the voltage space vectors. Then, judge whether the trigger mechanism is satisfied to determine whether it is necessary to switch the voltage space vector. Subsequently, predict the variation of the current error $\Delta i_{s}$ under the action of different voltage space vectors (*k* = 0–18). By judging the relationship between $\left|\Delta i_{s}\right|$ and $\left|\Delta i_{s\max}\right|$ at the future moment, poll and optimize to select a new voltage space vector. For details, refer to Fig 8, where the traditional voltage space vector optimization strategy can be given as (23):


$$\begin{array}{c} \left\{ \begin{array}{c} \Delta t(k_{\text{new}})\to \text{min}\\ \frac{\Delta t(k_{\text{new}})}{m(k_{\text{new}})}\to \max \end{array}\right. \end{array}$$ (23)

**Fig 8:**
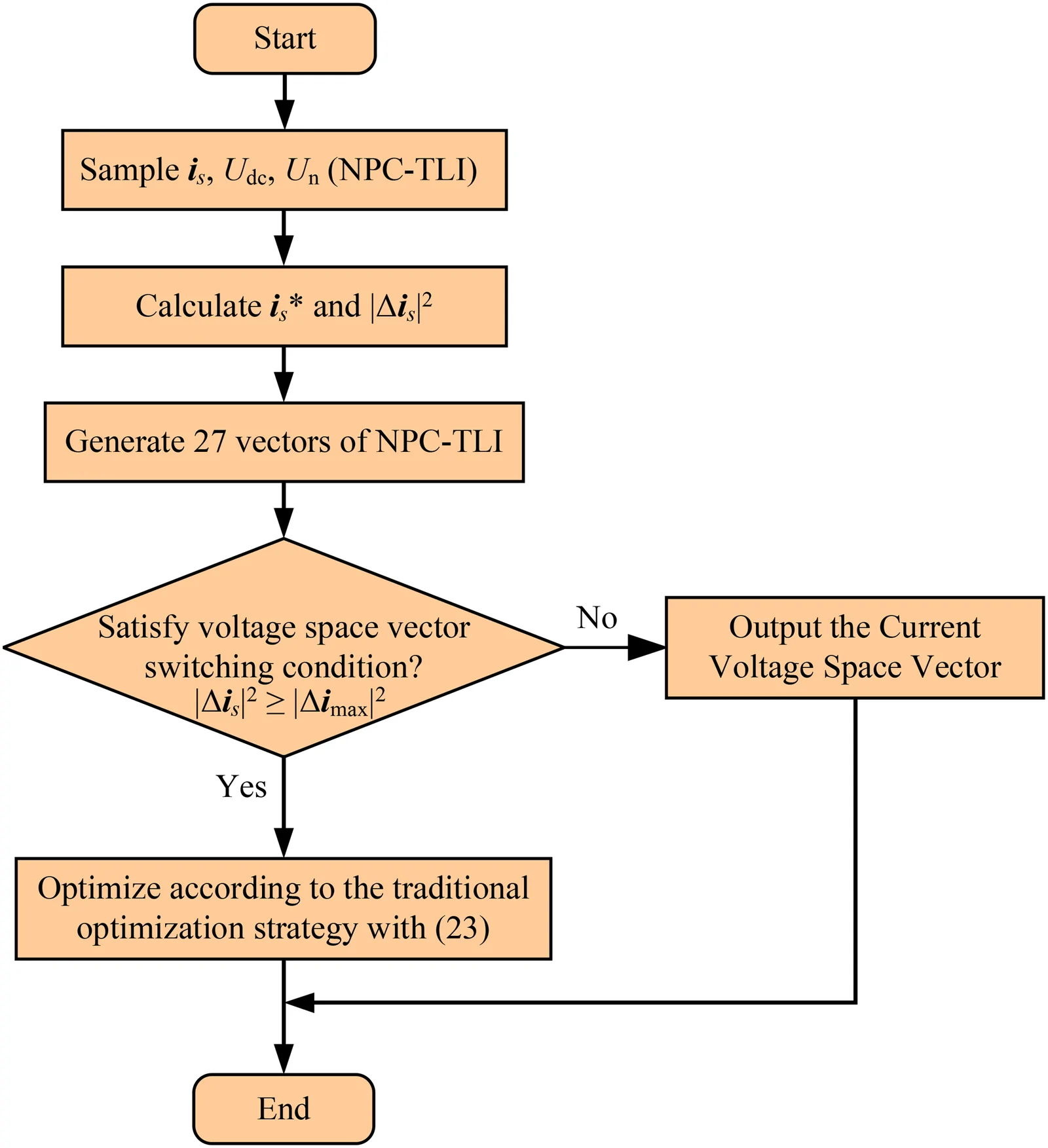
The Flow chart of traditional MPCC.

### Improvement of the model predictive current control strategy

In the context of long-cable PWM-driven IM ESP systems, however, the conventional control focus of traditional current circular trajectory MPCC introduces significant practical limitations. The high switching frequency resulting from the pursuit of fast current regulation not only causes excessive inverter switching losses but also triggers severe overvoltage transients along the long cable due to its distributed capacitance and inductance characteristics. This in turn exacerbates the voltage stress on power devices and undermines the operational stability of the entire drive system.

To address the above issues, the core objective of this study is to optimize the voltage space vector selection strategy of MPCC. An optimal voltage space vector $u_{sk}$ is selected to maximize the residence time of the stator current vector $i_{s}$ within a predefined circular boundary [33]. This improved design is specifically tailored for long-cable PWM-driven IM ESP systems, effectively mitigating cable overvoltage transients caused by frequent switching, reducing the switching frequency of power semiconductor devices, and minimizing associated inverter switching losses while maintaining basic current control performance.

By shifting the control focus from the rapid current correction of traditional MPCC to maximizing the current residence time within the circular boundary, the proposed improved MPCC scheme addresses the deficiencies of conventional methods in long-cable applications. The detailed design and implementation of this improved control strategy are elaborated in the subsequent sections.

Let *t*_0_ denote the current sampling instant, and *t* represent the instant after a time interval Δt has elapsed. The reference current vector at the future instant *t* is denoted as $i_{s}^{\text{ref}}(t)$. Under the action of *t*he *k*-th voltage space vector $u_{sk}$, the predicted stator current at the future instant *t* is $i_{s}(t,k)$. The future current deviation $\Delta i_{s}(t,k)$ is thus defined as (24):


$$\begin{array}{c} \Delta i_{s}(t,k)=i_{s}^{\text{ref}}(t)-i_{s}(t,k)=e(t_{0})+e^{\prime }(t_{0},k)\cdot \Delta t \end{array}$$ (24)

where *k* (*k* = 0–18) refers to the index of the voltage space vector, as illustrated in Fig 7 and (22); the reference and predicted current vectors can be expanded using a first-order Taylor series approximation about *t*_0_ (25):


$$\begin{array}{c} \left\{ \begin{array}{c} i_{s}^{\text{ref}}(t)=i_{s}^{\text{ref}}(t_{0})+{\frac{di_{s}^{\text{ref}}(\tau )}{d\tau }|}_{\tau =t_{0}}\cdot \Delta t\\ i_{s}(t,k)=i_{s}(t_{0})+{\frac{di_{s}(\tau ,k)}{d\tau }|}_{\tau =t_{0}}\cdot \Delta t \end{array}\right. \end{array}$$ (25)

where τ denotes the normalized time variable; $i_{s}^{\text{ref}}(t_{0})$ and $i_{s}(t_{0})$ represent the reference and predicted current vector at *t*_0_; ${\frac{di_{s}^{\text{ref}}(\tau )}{d\tau }|}_{\tau =t_{0}}$ describes the rate of change of the reference current vector, ${\frac{di_{s}(\tau ,k)}{d\tau }|}_{\tau =t_{0}}$ is the rate of change of the predicted actual current vec*t*or, which is dependent on the *k*-th voltage vector applied.

According to (1), one can get the derivative of stator current as (26).


$$\begin{array}{c} \frac{di_{s}}{dt}=\frac{1}{\tau_{\sigma }^{\prime }}\left[-j\omega_{s}\tau_{\sigma }^{\prime }i_{s}-i_{s}+\frac{K_{r}}{R_{\sigma }}\left(\frac{1}{\tau_{r}}-j\omega_{r}\right)\psi_{r}+\frac{1}{R_{\sigma }}u_{s}\right] \end{array}$$ (26)

By substituting the system state variables into the discrete model, the time-domain derivative of the stator current at time *t*_0_ under the action of voltage vector $u_{sk}$ can be explicitly expressed as (27).


$$\begin{array}{c} {\frac{di_{s}(\tau ,k)}{d\tau }|}_{\tau =t_{0}}=\frac{1}{\tau_{\sigma }^{\prime }}\left[{\left(-j\omega_{s}\tau_{\sigma }^{\prime }-1\right)i_{s}|}_{\tau =t_{0}}+{\frac{K_{r}}{R_{\sigma }}\left(\frac{1}{\tau_{r}}-j\omega_{r}\right)\psi_{r}|}_{\tau =t_{0}}+\frac{1}{R_{\sigma }}u_{sk}\right] \end{array}$$ (27)

It can be seen that (27) only involves the stator current vector $i_{s}{|}_{\tau =t_{0}}$ and rotor flux vector $\psi_{r}{|}_{\tau =t_{0}}$ at time *t*_0_, as well as the stator voltage vector $u_{sk}$ applied to the IM. No differen*t*ial operation is performed in the process of calculating the current derivative, i.e., the proposed model has better robustness and can reduce the adverse effects caused by measurement noise and electrical disturbances.

To determine the relationship between the current vector and the boundary circle at instant *t*, one can examine whether $\left|\Delta i_{s}(t,k)\right|$ is greater than $\left|\Delta i_{s\max}\right|$):

if $\left|\Delta i_{s}(t,k)|>|\Delta i_{s\max}\right|$, it indicates that $i_{s}(t)$ lies outside the boundary circle;
if $\left|\Delta i_{s}(t,k)|<|\Delta i_{s\max}\right|$, it indicates that $i_{s}(t)$ lies inside the boundary circle;
if $\left|\Delta i_{s}(t,k)|=|\Delta i_{s\max}\right|$, it indicates that $i_{s}(t)$ is exactly tangent to the boundary circle.

Since all variables are space vectors, one can calculate the magnitude of $i_{s}(t,k)$ by squaring its components, which yields a quadratic equation (28). Thus, the magnitude comparison between $\left|\Delta i_{s}(t,k)\right|$ and $\left|\Delta i_{s\text{max}}\right|$ can be transformed into a problem of solving the quadratic inequality in (29), i.e., solving their intersection point as (30):


$$\begin{array}{c} \left|\Delta i_{s}(t,k){|}^{2}=a(t_{0},k)\cdot \Delta t^{2}+b(t_{0},k)\cdot \Delta t+c(t_{0}\right) \end{array}$$ (28)

$$\begin{array}{c} |\Delta i_{s}(t,k){|}^{2}\leq |\Delta i_{s}\text{max}{|}^{2} \end{array}$$ (29)

$$\begin{array}{c} |\Delta i_{s}(t,k){|}^{2}-|\Delta i_{s\text{max}}{|}^{2}=a(t_{0},k)\cdot \Delta t^{2}+b(t_{0},k)\cdot \Delta t+c(t_{0})-|\Delta i_{s\text{max}}{|}^{2} \end{array}$$ (30)

where the coefficients *a*(*t*_0_,*k*), *b*(*t*_0_,*k*) and *c*(*t*_0_) are derived from the two components of each space vector as (31):


$$\begin{array}{c} \left\{ \begin{array}{c} a(t_{0},k)={e_{d}^{\prime }}^{2}+{e_{q}^{\prime }}^{2}\\ b(t_{0},k)=2(e_{d}e_{d}^{\prime }+e_{q}e_{q}^{\prime })\\ c(t_{0})=e_{d}^{2}+e_{q}^{2} \end{array}\right. \end{array}$$ (31)

where $e_{d}$, $e_{q}$, $e_{d}^{\prime }$ and $e_{q}^{\prime }$ are the *d*-axis, *q*-axis components of $e(t_{0})$ and $e^{\prime }(t_{0},k)$, which are defined as (32) and (33), respectively:


$$\begin{array}{c} \left\{ \begin{array}{c} e_{d}=i_{sd}^{\text{ref}}-i_{sd}\\ e_{q}=i_{sq}^{\text{ref}}-i_{sq} \end{array}\right. \end{array}$$ (32)

$$\begin{array}{c} \left\{ \begin{array}{c} e_{d}^{\prime }={\frac{di_{sd}^{\text{ref}}(\tau )}{d\tau }|}_{\tau =t_{0}}-{\frac{di_{sd}(\tau ,k)}{d\tau }|}_{\tau =t_{0}}\\ e_{q}^{\prime }={\frac{di_{sq}^{\text{ref}}(\tau )}{d\tau }|}_{\tau =t_{0}}-{\frac{di_{sq}(\tau ,k)}{d\tau }|}_{\tau =t_{0}} \end{array}\right. \end{array}$$ (33)

From (30), it can be seen that the right root of this quadratic function represents the duration for which the current trajectory can remain within the circular boundary, and the basic voltage vector corresponding to the quadratic function curve that yields the maximum right root is the optimal voltage vector. This voltage vector optimization method directly alters the basic voltage space vector, allowing the optimal voltage space vector to act on the IM directly without a reference voltage vector modulation process. Thus, while reducing the switching frequency of the long-cable PWM-driven system, it still enables a rapid current response.

Refer to Fig 7, if the current vector ${𝑖}_{s}$ touches the boundary circle at time *t*_0_, it is necessary to change the curren*t* switching state. After the 19 voltage space vectors ${𝑢}_{sk}$ generated by the inverter act on the motor, the changing trends $d{𝑖}_{s}(\tau ,k)/d\tau$ of the current vector are different. Both the amplitude and moving direction of the stator current vector ${𝑖}_{s}$ will change accordingly, resulting in 19 change curves of current error. According to (30), these curves are all parabolas, as shown in Fig 9.

**Fig 9:**
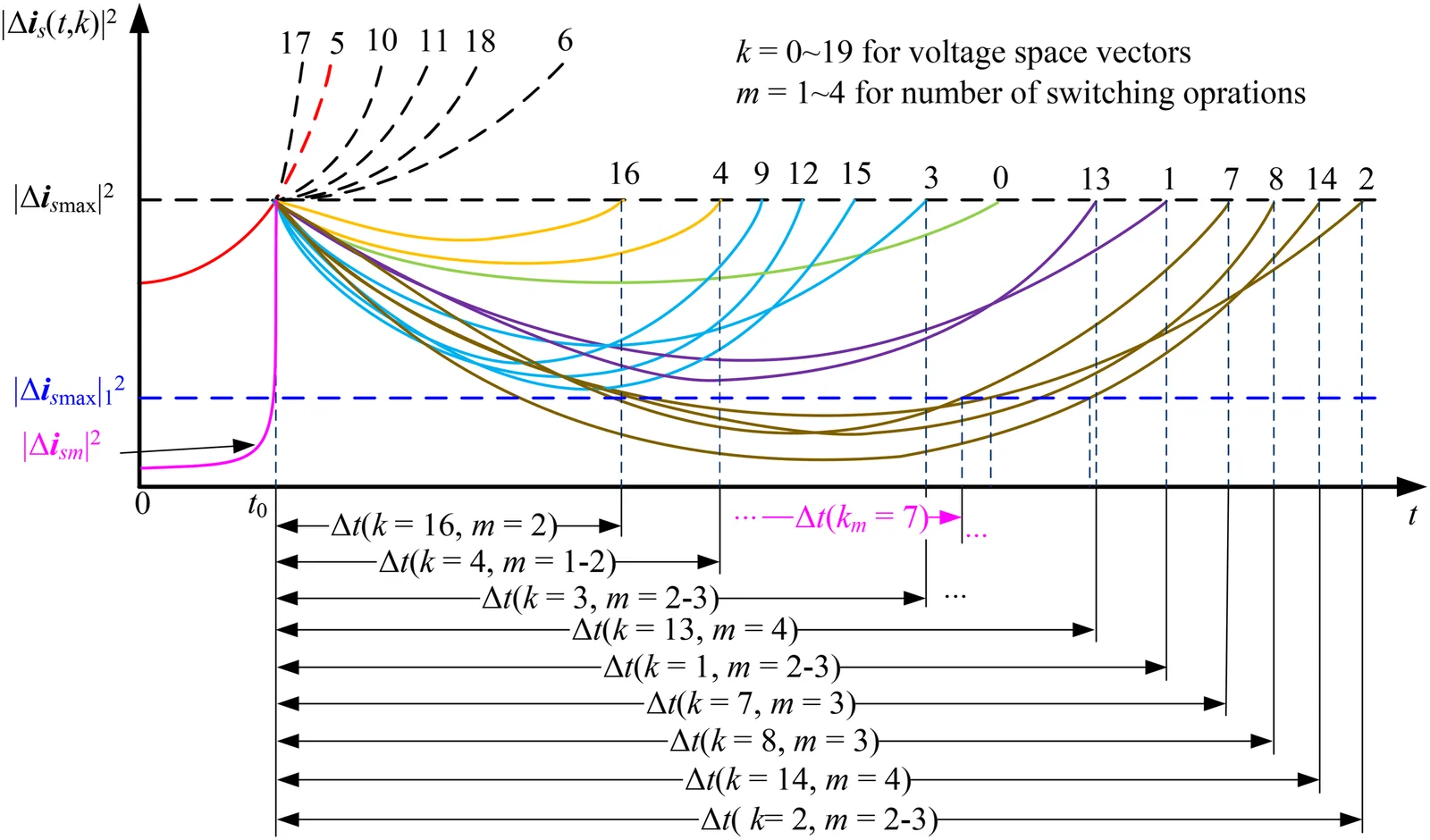
The current error graph with different switching state.

Assume that the current switching state is *k* = 5 (only the P-type combination OOP is considered; the N-type NNO is excluded); at time *t*_0_, curve 5 reaches $|\Delta i_{s\max}{|}^{2}$ and touches the boundary circle. If the current switching state *k* remains unchanged, the current error will continue to increase, as shown by the dashed part of curve 5 above $|\Delta i_{s\max}{|}^{2}$) in Fig 9. At this point, it is necessary to change the switching state of the inverter and select an optimal switching state from the other 18 switching states. To minimize the switching frequency of power devices, the voltage space vector $u_{sk\text{new}}$ generated by the new switching state *k*_new_ should keep the current vector $i_{s}$ inside the boundary circle as long as possible. Taking Fig 9 as an example, according to the above rules, the $u_{sk\text{new}}(k_{\text{new}}=17,10,11,18,6)$ will lead to the current $i_{s}$ breaching the specified circular boundary and hence be eliminated from the candidate set; for the other voltage space vectors, i.e., $u_{sk\text{new}}(k_{\text{new}}=16,4,9,12,15,3,0,13,1,7,8,14,2)$, the $u_{sk\text{new}}(k_{\text{new}}=2)$ is obviously optimal, because under its action, the time Δt required for the current error to reach the maximum again is the longest, that is, Δt(k=2)→max.

However, to reduce the switching frequency, the switching behavior of each power device must also be taken into account. The number of switching operations of all power devices during the transition of the inverter from the current state *k* to the new state *k*_new_ is denoted as *m*(*k*_new_). The optimal switching state *k*_new_ should ensure the longest duration Δt while also considering the number of switching operations *m*(*k*_new_). Therefore, the criterion for determining the optimal switching state as (34):


$$\begin{array}{c} \left\{ \begin{array}{c} \Delta t(k_{\text{new}})\to \text{max}\\ \frac{\Delta t(k_{\text{new}})}{m(k_{\text{new}})}\to \max \end{array}\right. \end{array}$$ (34)

Furthermore, if the reference speed $\omega_{r}^{\text{ref}}$ increases abruptly as Fig 5, the reference current vector $i_{s}^{\text{ref}}$ will experience a large mutation, which in turn causes the current vector $i_{s}$ to deviate from and move far away from the circle boundary instantaneously, leading to a sharp increase in the current error $|\Delta i_{s}(t,k){|}^{2}$. At this point, the primary task is to make the current vector return to the boundary circle as soon as possible, so the new switching state can no longer be selected according to (34).

As shown in Fig 9, at time *t*_0_, if the current error $|\Delta i_{sm}{|}^{2}$ (*m* represents mutation current) changes abruptly and greatly exceeds the allowable maximum value $|\Delta i_{s\max}{|}_{1}^{2}$. To quickly return the current vector to the interior of the boundary circle, the current error must be reduced rapidly. It can be clearly seen that the $\Delta t(k_{m}=7,8,14)$ are all shorter than that of $\Delta t(k_{m}=2)$, i.e., the states $k_{m}=7$ can reduce the current error in a short time. Therefore, the optimal switching states at this time are $k_{m}=7$ instead of the previous criterion (34), then a new criterion for determining the optimal switching state with current mutation is changed to (35).


$$\begin{array}{c} \Delta t(k_{m})\to \min \end{array}$$ (35)

As shown in Fig 6, the DC side consists of two series-connected identical capacitors with the neutral-point node denoted as O, then the neutral-point voltage deviation is defined as (36):


$$\begin{array}{c} \Delta u_{\text{O}}=u_{\text{O}}-\frac{U_{dc}}{2} \end{array}$$ (36)

Let the switching state of phase *x* be $S_{x}\in \{1,0,-1\}$ for {P, O, N}, where x∈{a,b,c}, corresponding to the output voltage levels {P, O, N}, respectively. Define the neutral-point status indicator function $X_{x}=1$ (when $S_{x}=0$) or 0 (when $S_{x}=\pm 1$). The current flowing into the neutral-point node O can then be expressed as (37):


$$\begin{array}{c} i_{\text{O}}=X_{a}i_{a}+X_{b}i_{b}+X_{c}i_{c} \end{array}$$ (37)

When both DC-side capacitors have the same capacitance *C*, the predicted neutral-point voltage deviation at the next sampling instant can be obtained via forward Euler discretization as (38):


$$\begin{array}{c} \Delta u_{\text{O}}(k+1)=\Delta u_{\text{O}}(k)+\frac{T_{s}}{2C}i_{\text{O}}(k) \end{array}$$ (38)

where $T_{s}$ is the sampling period, *C* is the DC-link capacitance, and *i*_O_ is the neutral-point current flowing into the neutral point O. As indicated by (38), the variation of $\Delta u_{\text{O}}$ is determined solely by the neutral-point current *i*_O_. Therefore, *i*_O_ is the only physical means to change the neutral-point voltage deviation. Only when $i_{\text{O}}\neq 0$ can $\Delta u_{\text{O}}$ be actively regulated.

For the *i*-th candidate voltage vector, the predicted increment of the neutral-point voltage deviation is given by (39):


$$\begin{array}{c} \Delta u_{\text{O},i}=\frac{T_{s}}{2C}i_{\text{O},i} \end{array}$$ (39)

where *i*_O,*i*_ is the neutral-point current corresponding to the *i*-th candidate state. As illustrated in Table 1, small vectors possess two redundant switching states (P-type and N-type) that generate identical output voltages but produce opposite neutral-point currents. The controller evaluates both redundant states and selects the one that yields the smaller $\Delta u_{\text{O}}(k+1)$, provided that the current tracking error remains within an acceptable tolerance. In this way, the neutral-point balancing mechanism is activated only when the candidate vector belongs to the small-vector group, ensuring active neutral-point voltage regulation without compromising the current control performance.

Still taking Fig 9 as an example, assume that the current operating state corresponds to *k* = 5 (OOP), *u*_O_(*k* = 5) increases. If the current error minimization criterion in (34) selects *k*_new_ = 2 as the candidate voltage vector to reduce the current tracking error, then the N-type redundant state OON should be adopted. This choice not only maximizes the residence time of the current trajectory within the circular boundary, but also enables active regulation of the neutral-point voltage, i.e., increasing $u_{\text{O}}(k_{\text{new}}=2)$ to maintain the neutral-point voltage balance. On the other hand, if the voltage vector selection criterion in (35) yields *k*_new_ = 7, then only the medium vector PON is available as the selected switching combination, since medium vectors do not possess redundant switching states for neutral-point voltage regulation.

Based on the above analysis, one can obtain the flow chart of improved MPCC as Fig 10. Where, the proposed improved MPCC strategy employs a dual-mode cost function based on a circular boundary constraint. The normal cost function *J*_in_ (40) is used when the predicted current error lies within the circular boundary, while the backup cost function *J*_out_ (41) is activated when the current error exceeds the boundary.


$$\begin{array}{c} J_{\text{in}}=\|u_{i}-u^{\text{ref}}{\|}^{2}+\lambda_{\text{sw}}\cdot \Delta n_{\text{sw}}+\lambda_{\text{du}}\cdot \|u_{i}-u_{\text{prev}}{\|}^{2} \end{array}$$ (40)

$$\begin{array}{c} J_{\text{out}}=\|\Delta i_{s}(k+1){\|}^{2}+\lambda_{\text{bu}}\cdot \|u_{i}-u^{\text{ref}}{\|}^{2}+\lambda_{\text{sw}}\cdot \Delta n_{\text{sw}}+\lambda_{\text{du}}\cdot \|u_{i}-u_{\text{prev}}{\|}^{2} \end{array}$$ (41)

where, $u_{i}$ is the *i*-th (*i* = 0–18) candidate voltage vector from the finite control set (19 basic vectors); $u^{\text{ref}}$ is the Deadbeat reference voltage computed from the motor model and current errors; $u_{\text{prev}}$ is the voltage vector applied in the previous sampling period; $\Delta n_{\text{sw}}$ denotes the number of switching transitions between $u_{i}$ and $u_{\text{prev}}$; $\Delta i_{s}(k+1)$ is the predicted current error at the next sampling instant; $\lambda_{\text{sw}}=0.008$ is the weighting factor for switching frequency penalty; $\lambda_{\text{du}}=0.03$ is the weighting factor for *du*/*dt* suppression; and $\lambda_{\text{bu}}=0.2$ is the weighting factor for voltage tracking error in the backup cost.

**Fig 10:**
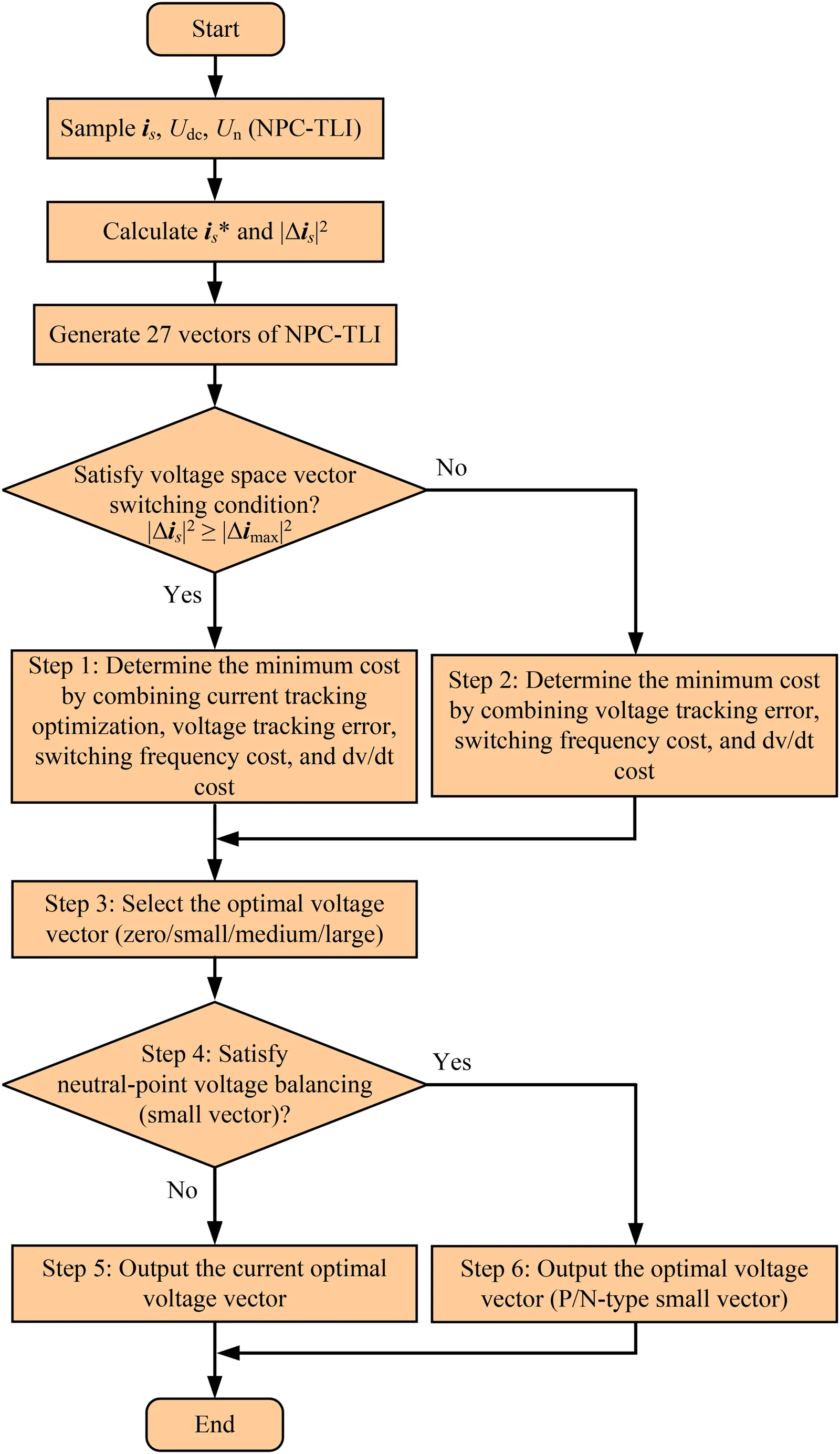
The Flow chart of improved model predictive current control.

In (40), the first term $\|u_{i}-u^{\text{ref}}{\|}^{2}$ ensures voltage tracking accuracy by minimizing the deviation between the candidate vector and the Deadbeat reference. The second term $\lambda_{\text{sw}}\cdot \Delta n_{\text{sw}}$ penalizes switching transitions to reduce switching losses. The third term $\lambda_{\text{du}}\cdot \|u_{i}-u_{\text{prev}}{\|}^{2}$ suppresses *du*/*dt* by minimizing the voltage jump between consecutive switching periods, thereby mitigating overvoltage in long-cable drives.

In (41), the first term $\|\Delta i_{s}(k+1){\|}^{2}$ is given the highest priority to quickly drive the current trajectory back into the circular boundary during transients. The second term is weighted by a reduced factor $\lambda_{\text{bu}}=0.2$, which lowers the priority of voltage tracking when the current error is excessive. The third and fourth terms remain identical to those in (40) to maintain switching loss reduction and *du*/*dt* suppression during recovery.

The optimal voltage vector is selected according to the following rule: if at least one candidate vector satisfies the boundary constraint (i.e., the cost from (40) is available), the vector corresponding to the minimum *J*_in_ is selected. Otherwise, the vector corresponding to the minimum *J*_out_ is chosen as the final output. This dual-mode mechanism enables the proposed strategy to achieve high steady-state efficiency while maintaining fast dynamic response during transients.

By comparing Figs 8 and 10, the fundamental difference between the traditional MPCC and the improved MPCC lies in the voltage vector optimization criterion. The improved scheme introduces a comprehensive cost function that combines current tracking optimization, voltage tracking error, switching frequency cost, and *du*/*dt* cost. The voltage vector that minimizes this comprehensive cost is then selected as the optimal output. Subsequently, if the selected vector is a small vector, the neutral-point voltage balancing criterion is further applied: the redundant state (P-type or N-type) that helps suppress the neutral-point voltage deviation is selected. Through this hierarchical optimization structure, the improved MPCC effectively reduces switching frequency and *du*/*dt*, thereby suppressing overvoltage and maintaining neutral-point voltage balance in long-cable PWM-driven systems.

## Simulation analysis and experimental verification

To validate the effectiveness of the proposed improved MPCC strategy, the simulation and experimental validations are carried out in three complementary stages. First, a 1 km long-cable simulation with an RLC filter is conducted to assess the overvoltage suppression performance under typical deep-well ESP operating conditions, where severe voltage reflection effects are expected. Second, a 110 m long-cable simulation without an RLC filter is performed to establish a direct comparative baseline for the subsequent experimental validation. This configuration is intentionally chosen to match the experimental setup exactly, enabling a one-to-one comparison between simulation and experiment. Third, experimental tests are carried out under the identical 110 m no-filter configuration to validate the simulation model and verify the practical effectiveness of the proposed control strategy. The corresponding results for the three stages are presented in the following sections.

The parameters of long-cable transmission line as #4 ~ AWG, $R_{0}=0.98\;\Omega$/km, *L*_0_ = 0.4597 mH/km, $C_{0}=0.2626\;\mu$F/km; the IM parameters and rated values are shown in Table 2.

**Table 2:** The parameters of induction motor.

Parameters	Values
Rated power (kW)	*P*_N_ = 32
Rated voltage (V)	*U*_N_ = 900
Rated current (A)	*I*_N_ = 37
Rated frequency (Hz)	*f*_N_ = 50
Rated speed (r/min)	*n*_N_ = 2900
Stator/Rotor resistance (Ω)	$R_{s}=0.768$, $R_{r}=0.778$
Stator/Rotor inductance (mH)	$L_{s}=4.544$, $L_{r}=5.814$
Mutual inductance (mH)	$L_{m}=23$
Pole pairs	*p* = 1
Moment of inertia (kg·m^2^)	*J* = 0.0392

The cable line characteristic impedance as $Z_{0}=\sqrt{L_{0}/C_{0}}=41.84\;\Omega$, the equivalent impedance of IM is $Z_{L}=0.44+j2.86\Omega$. Set the cable length to 1 km, then the main parameters of RLC filter as: the damping resistor $R_{f}$ is determined by $R_{f}=Z_{0}$ and $R_{f}=2\zeta \sqrt{L_{f}/C_{f}}$ to realize impedance matching with the cable impedance and adjust the system damping ratio, here two damping ratios are adopted, where ζ=1.31 (over-damped) yields $R_{f}=Z_{0}\approx 42\;\Omega$ and ζ=0.707 (under-damped) yields $R_{f}=22\;\Omega$. And set the cutoff frequency $f_{n}=1$ kHz, natural angular frequency $\omega_{n}=2\pi f_{n}$, referring to (21), then the filter capacitance and inductance as $C_{f}=10\;\mu$F, $L_{f}=2.53$ mH, respectively.

The equivalent switching frequency *f*_eq_ used in the following analysis is defined as the total number of switching transitions of all power semiconductor devices within a fixed time window, divided by the window duration. This metric is computed using a fixed-window averaging method:


$$\begin{array}{c} f_{\text{eq}}=\frac{N_{\text{sw}}}{T_{\text{window}}} \end{array}$$ (42)

where *T*_window_ = 0.1 s is the fixed statistical window duration, and *N*_sw_ is the total number of switching transitions of all IGBTs within this window. The switching transition count is calculated as:


$$\begin{array}{c} \Delta n_{k}=|S_{a}(k)-S_{a}(k-1)|+|S_{b}(k)-S_{b}(k-1)|+|S_{c}(k)-S_{c}(k-1)| \end{array}$$ (43)

where $S_{a},S_{b},S_{c}$ are the switching states of the three phases. This fixed-window averaging method yields smooth frequency curves because it represents the average switching frequency over each 0.1 s window, effectively suppressing the high-frequency fluctuations inherent in the non-uniform switching behavior of MPCC.

### Simulation analysis of the PWM-driven IM ESP system

#### Frequency response simulation of RLC filter.

Substituting the above two sets of parameters into the RLC transfer function given in (18), the corresponding Bode plot of zero-point-included second-order RLC filter as shown in Fig 11. Where the magnitude and phase responses are obtained at a cutoff frequency of 1 kHz under two damping ratios: ζ = 1.31 (over-damped, solid blue line) and ζ = 0.707 (near-critically damped, dashed red line). The magnitude response (upper) in decibels (dB) shows effective low-pass filtering characteristics, while the phase response (lower) in degrees (deg) illustrates the phase shift variation with frequency. The over-damped case yields a smoother roll-off without resonance, whereas the near-critically damped case provides steeper attenuation and stronger harmonic suppression. These results demonstrate that the damping ratio significantly affects the filtering performance and dynamic response of the RLC filter.

**Fig 11:**
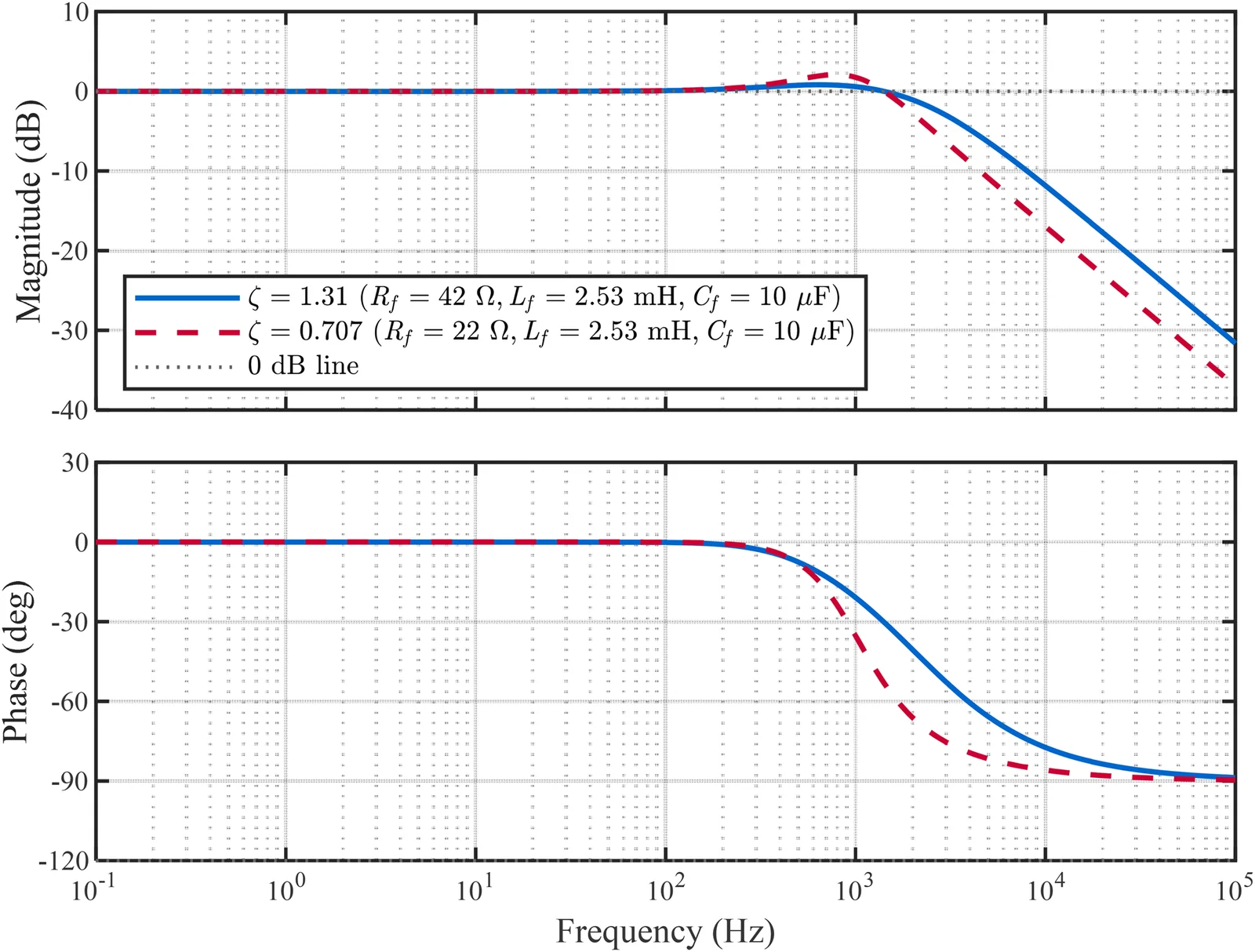
Bode plot of zero‑point‑included second‑order RLC filter.

#### Simulation analysis of 1 km cable system with an RLC filter.

To mitigate cable-induced reflected overvoltage and LC resonance during experiments, an RLC filter is applied. It should be noted that two sets of RLC filter parameters are considered in this work. The first set ($L_{f}=2.53\text{ mH}$, $C_{f}=10\;\mu \text{F}$, above mentioned) is a theoretical design based on a 1 kHz cutoff frequency, which serves as a reference to illustrate the analytical filter design methodology. The second set ($L_{f}=25\;\text{mH}$, $C_{f}=60\;\mu \text{F}$, $R_{f}=20\;\Omega$) is the engineering-adjusted configuration used in the actual experiments and simulations under the improved MPCC scheme. This adjustment is made based on the equivalent switching frequency of the improved MPCC obtained from subsequent simulations (approximately 760 Hz under the 1 km cable condition). Following the empirical design principle that the filter cutoff frequency should be approximately one-tenth to one-fifth of the switching frequency, the ratio $f_{c}/f_{sw}\approx 0.17$ falls within this typical engineering range. The enlarged inductor enhances high-frequency harmonic attenuation, while matched resistance increases damping to restrain resonant voltage fluctuations and safeguard motor insulation. Its reduced resonant frequency eliminates harmonic amplification around switching frequencies, enabling stable and reliable performance of long-cable ESP induction motor drives. The adjusted parameters are recommended for industrial applications.

Simulation parameters are configured as follows: the system initiates at 0.1 s with a reference motor speed of 0.8 p.u.; full rated load torque (105 N·m) is applied at 0.5 s; the radius of current error boundary circle is set to 0.2 p.u.; DC-link voltage equals 1300 V; the SVPWM scheme operates at a 2 kHz switching frequency and 20 kHz sampling frequency.

The simulation results are shown in Figs 12–16, respectively. Where, Fig 12 compares the motor speed and equivalent switching frequencies. As illustrated in Fig 12(a), 12(c) and 12(e), under the three control strategies, the motor speed can all track the reference value of 0.8 p.u; the maximum speed deviation of the traditional and improved MPCC are all approximately 0.07 p.u., and their steady-state deviation are about 0.01–0.02 p.u; while the SVPWM strategy achieves slightly better characteristics, maximum speed deviation of 0.03 p.u. In Fig 12(b) and 12(d), for the traditional MPCC, the maximum equivalent switching frequency reaches 4600 Hz during the startup stage (0.1–0.3 s), in the steady-state operation period (0.6–1 s), its equivalent switching frequency ranges from 765 Hz to 1000 Hz with an average value of 862.8 Hz; for the improved MPCC, the peak equivalent switching frequency is 3720 Hz within startup stage (0.1–0.3 s), and varies between 720 Hz and 800 Hz with an average of 760 Hz in steady state (0.6–1 s). As a result, in steady-state operation, the improved MPCC strategy has the lowest equivalent switching frequency, which is approximately 88% of that for traditional MPCC and 38% of SVPWM strategy (2 kHz), thereby contributing to lower switching losses.

**Fig 12:**
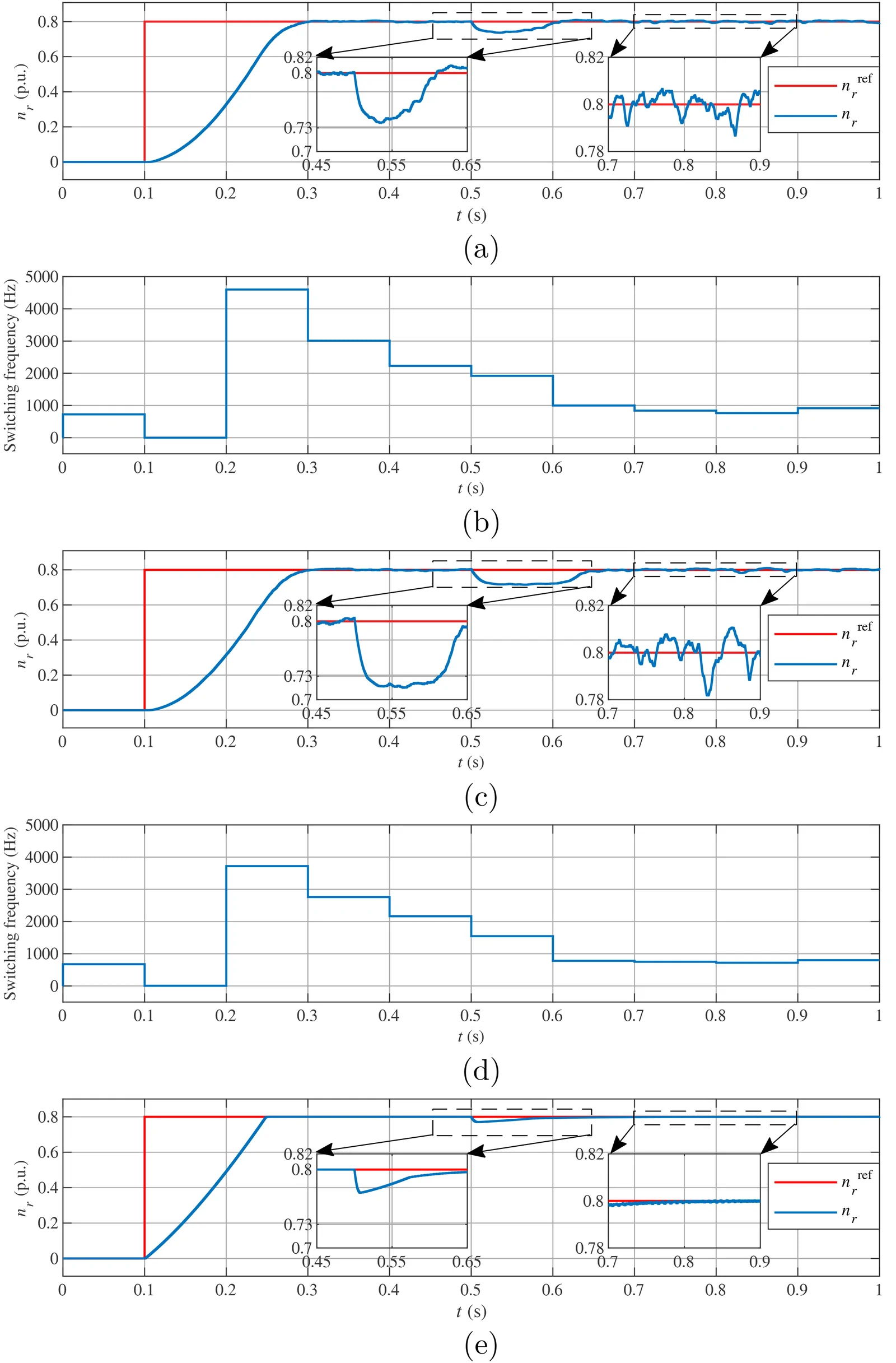
Simulation results of motor speed with zoomed-in views and equivalent switching frequency under 1 km long-cable PWM-driven system. (a) Speed for traditional MPCC. (b) Switching frequency for traditional MPCC. (c) Speed for improved MPCC. (d) Switching frequency for improved MPCC. (e) Speed for SVPWM.

**Fig 13:**
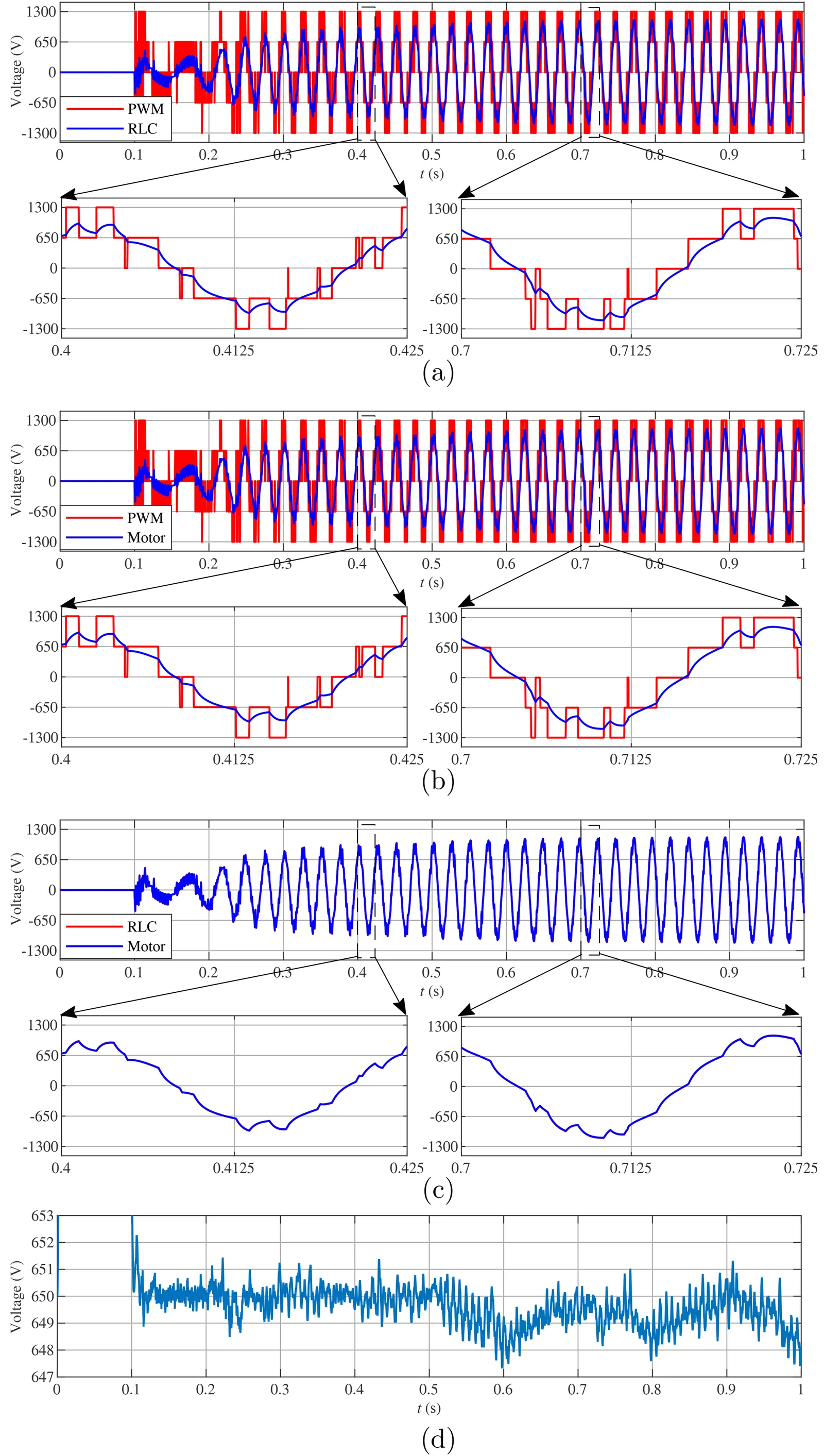
Simulation results of voltages in the traditional MPCC 1 km long-cable PWM-driven system. (a) Inverter and RLC filter output voltages with zoomed-in views. (b) Inverter and motor voltages with zoomed-in views. (c) RLC filter output and motor voltages with zoomed-in views. (d) Neutral-point voltage.

**Fig 14:**
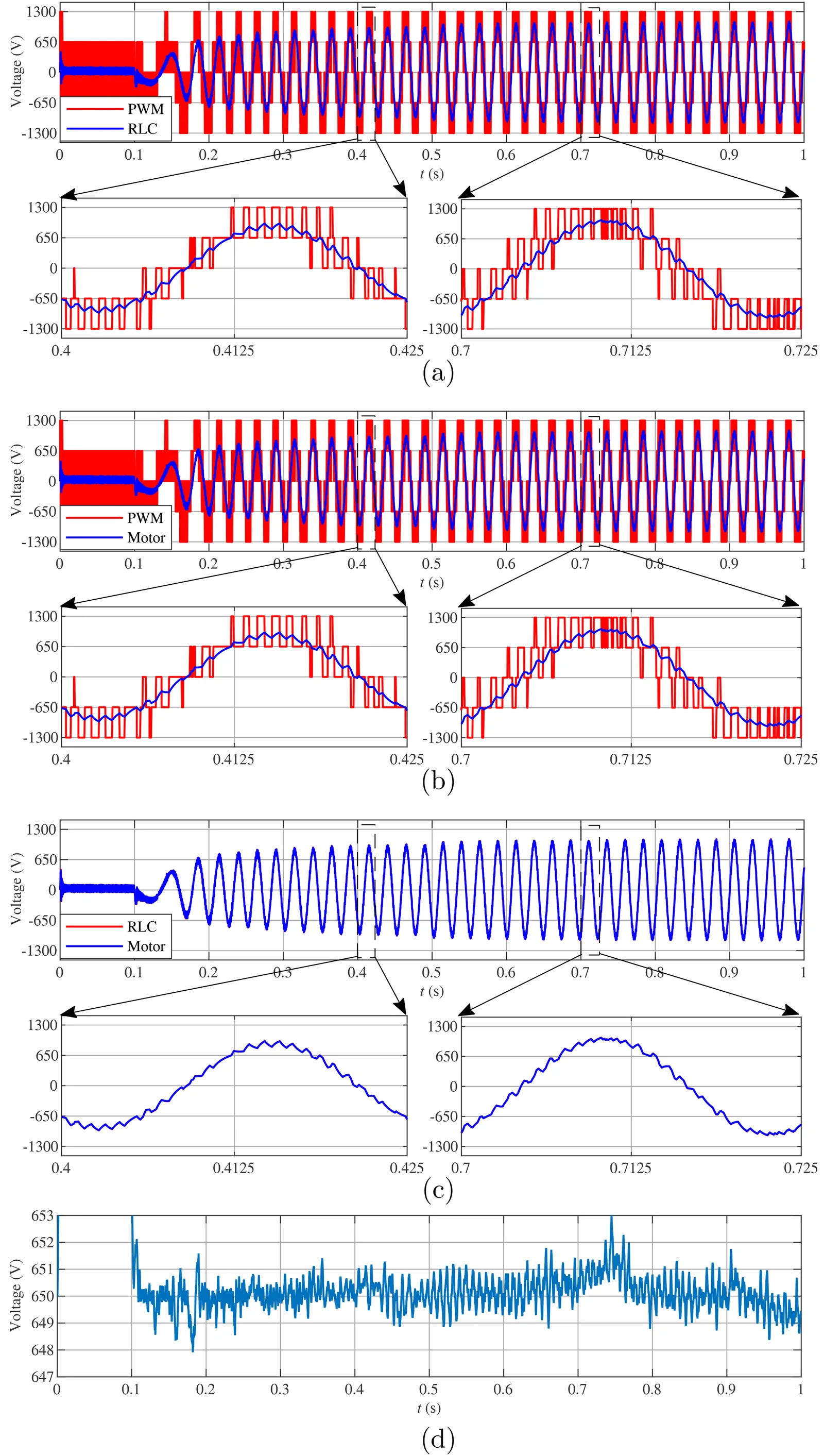
Simulation results of voltages in the improved MPCC 1 km long-cable PWM-driven system. (a) Inverter and RLC filter output voltages with zoomed-in views. (b) Inverter and motor voltages with zoomed-in views. (c) RLC filter output and motor voltages with zoomed-in views. (d) Neutral-point voltage.

**Fig 15:**
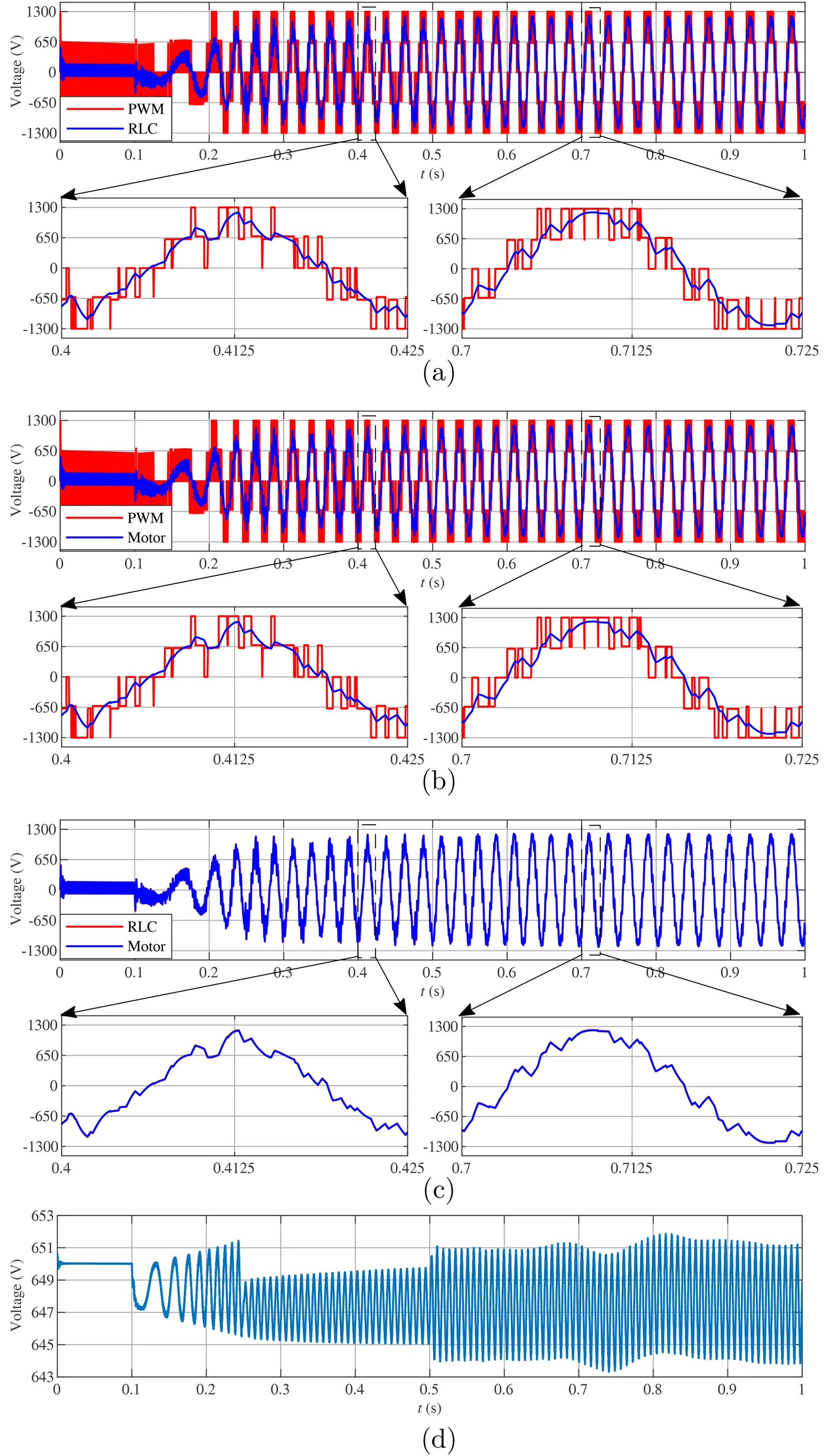
Simulation results of voltages in the SVPWM-driven 1 km long-cable system. (a) Inverter and RLC filter output voltages with zoomed-in views. (b) Inverter and motor voltages with zoomed-in views. (c) RLC filter output and motor voltages with zoomed-in views. (d) Neutral-point voltage.

**Fig 16:**
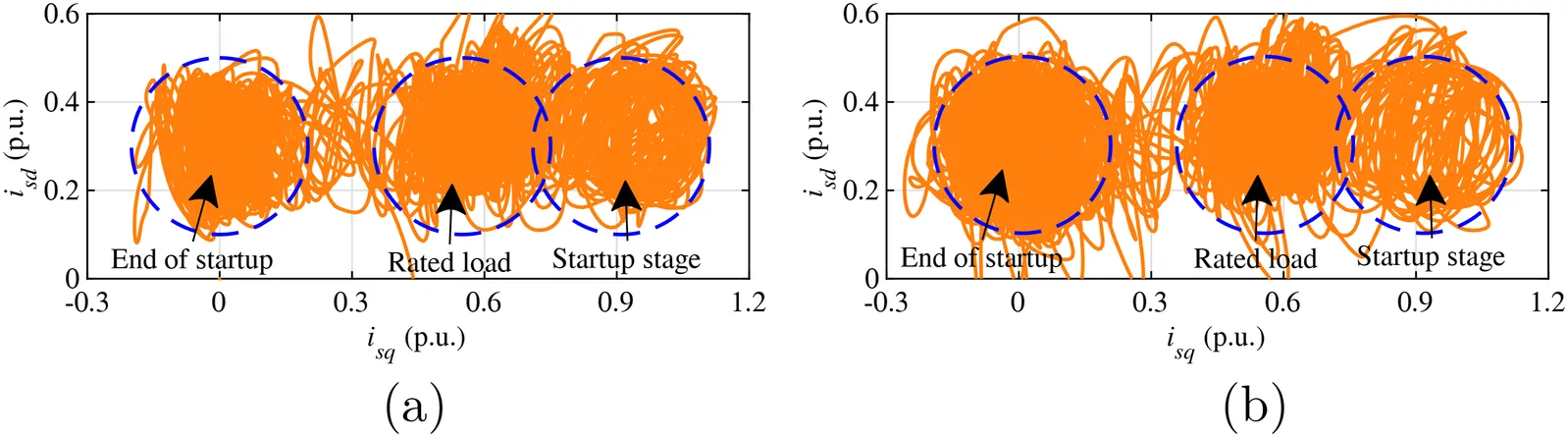
Simulation results of the stator d-q current trajectory with circular boundary predictive control in 1 km long-cable PWM-driven system. (a) Traditional MPCC. (b) Improved MPCC.

Figs 13–15 compare the output voltages of NPC-TLI, RLC filter, motor terminal with their zoomed-in views and neutral-point voltages under the traditional MPCC, improved MPCC and SVPWM strategies. First, as shown in Figs 13–15(a), 15(b) and 15(c), the suppression effect of the RLC filter is effectively demonstrated across all three control strategies, the high-frequency spikes in the inverter output PWM voltage are significantly attenuated after passing through the RLC filter, and the waveforms become smoother. This indicates that the basic filtering function of the RLC filter depends primarily on its own parameter design rather than on the control strategy, with similar filtered voltage quality observed across all three strategies. Second, the overvoltage stress after long-cable transmission is significantly improved (subplots (b) of Figs 13–15). After the PWM voltage is filtered by the RLC filter and transmitted through the 1 km long cable to the motor terminal, the overvoltage spikes are reduced under all three control strategies. Under the SVPWM strategy, noticeable overvoltage spikes remain at the motor terminal; under the traditional MPCC strategy, these overvoltage spikes are somewhat mitigated; under the improved MPCC strategy, the motor terminal voltage waveform is the smoothest with minimal overvoltage spikes, demonstrating that the improved MPCC effectively reduces high-frequency excitation components at the source by optimizing the voltage vector switching path. Third, the RLC-filtered voltage and motor terminal voltage waveforms are quite close to each other (subplots (c) Figs 13–15).

Under all three control strategies, the filtered voltage and motor terminal voltage waveforms largely overlap, with only minor amplitude differences. This indicates that at a cable length of 1 km, the long cable primarily introduces a small voltage drop. A comprehensive comparison of the motor terminal overvoltage values (with respect to the rated voltage of 900 V) reveals the following: the peak voltage of motor terminal under traditional MPCC is 1135.4 V (1.26 p.u.), under improved MPCC is the lowest at 1090.4 V (1.21 p.u.), and under SVPWM is 1216.6 V (1.35 p.u.). Thus, compared with traditional MPCC and SVPWM strategies, the improved MPCC achieves reductions of 4.0% and 10.4%, respectively. The safety margins with respect to the safe voltage limit (IEC 60034-18-41: 2014, 1836 V) are 700.6 V, 745.6 V, and 619.4 V, respectively. The improved MPCC strategy provides the largest voltage safety margin, and under all three control strategies, motor insulation safety is not compromised (according to IEC 60034-18-41: 2014, 1350 V).

The underlying mechanism for this improvement can be explained as follows. The overvoltage suppression of the improved MPCC stems from two mechanisms. First, the cost function penalizes switching transitions and voltage jumps, which inherently eliminates narrow pulses that contain rich high-frequency components capable of exciting cable reflections. Second, the circular boundary constraint ensures smooth voltage vector transitions, actively avoiding the double-pulsing phenomenon where consecutive switching events occur before the previous oscillation decays. Both mechanisms reduce the high-frequency excitation of traveling wave reflections without altering the fixed physical parameters of the cable and motor.

Figs 13–15(d) present the neutral-point voltage waveforms under three different control strategies: for the traditional MPCC, the neutral-point voltage fluctuates within 647.4–653.9 V with an average value of 649.6 V; for the improved MPCC strategy, the neutral-point voltage varies from 647.9–653.9 V and has an average value of 650.1 V; in terms of the SVPWM strategy, its neutral-point voltage ranges between 643.3–651.9 V, with an average value of 647.6 V. As a result, the improved MPCC features smaller neutral-point voltage fluctuations and keeps the operating voltage closer to the reference value of 650 V.

Fig 16 shows the stator current trajectories in the $i_{d}$-$i_{q}$ plane under traditional and improved MPCC strategies, indicating that both methods can limit the current error within a certain range.

In summary, as illustrated in Table 3, all three control strategies, in conjunction with the RLC filter, effectively suppress long-cable overvoltage and satisfy the insulation safety requirements at a cable length of 1 km. Among them, the improved MPCC strategy exhibits the lowest overvoltage peak, the largest safety margin, the best waveform quality, and the smallest neutral-point voltage fluctuation. Its active optimization of the voltage vector switching path achieves optimal synergy with the passive filtering of the RLC filter. Although at a cable length of 1 km, the traditional MPCC and SVPWM strategies also ensure motor insulation safety, the advantages of the improved MPCC strategy in overvoltage suppression and equivalent switching frequency reduction become increasingly prominent as the cable length further increases and the reflection effect intensifies. This fully demonstrates its technical superiority and engineering application value in long-cable PWM-driven ESP systems.

**Table 3:** Comparison of simulation result characteristics for traditional MPCC, improved MPCC and SVPWM strategies in 1 km cable system.

Key metric	Traditional MPCC	Improved MPCC	SVPWM
Peak voltage of motor terminal (V)	1135.4	1090.4	1216.6
Overvoltage ratio (relative to 900 V)	1.26	1.21	1.35
Safety voltage margin (V) (relative to 1836)	700.6	745.6	619.4
Duration beyond limit voltage (>1836 V)	0%	0%	0%
Duration beyond high‑stress region (>1350 V)	0%	0%	0%
Neutral-point voltage (V)	647.4–653.9 (mean 649.6)	647.9–653.9 (mean 650.1)	643.3–651.9 (mean 647.6)
Equivalent switching frequency (Hz) (steady-state: 0.6–1 s)	765.0–1000.0 (mean 862.8)	720.0–800.0 (mean 760.0)	2000

#### Simulation analysis of 110 m cable system without an RLC filter.

The corresponding results for the 110 m no-filter simulation are presented in Figs 17–21, where the motor speed and its reference value, equivalent switching frequencies are served as a direct cross-validation baseline for the experimental findings, while the above mentioned 1 km simulation with RLC filter examines the scalability of proposed MPCC strategy under more demanding long-cable conditions.

**Fig 17:**
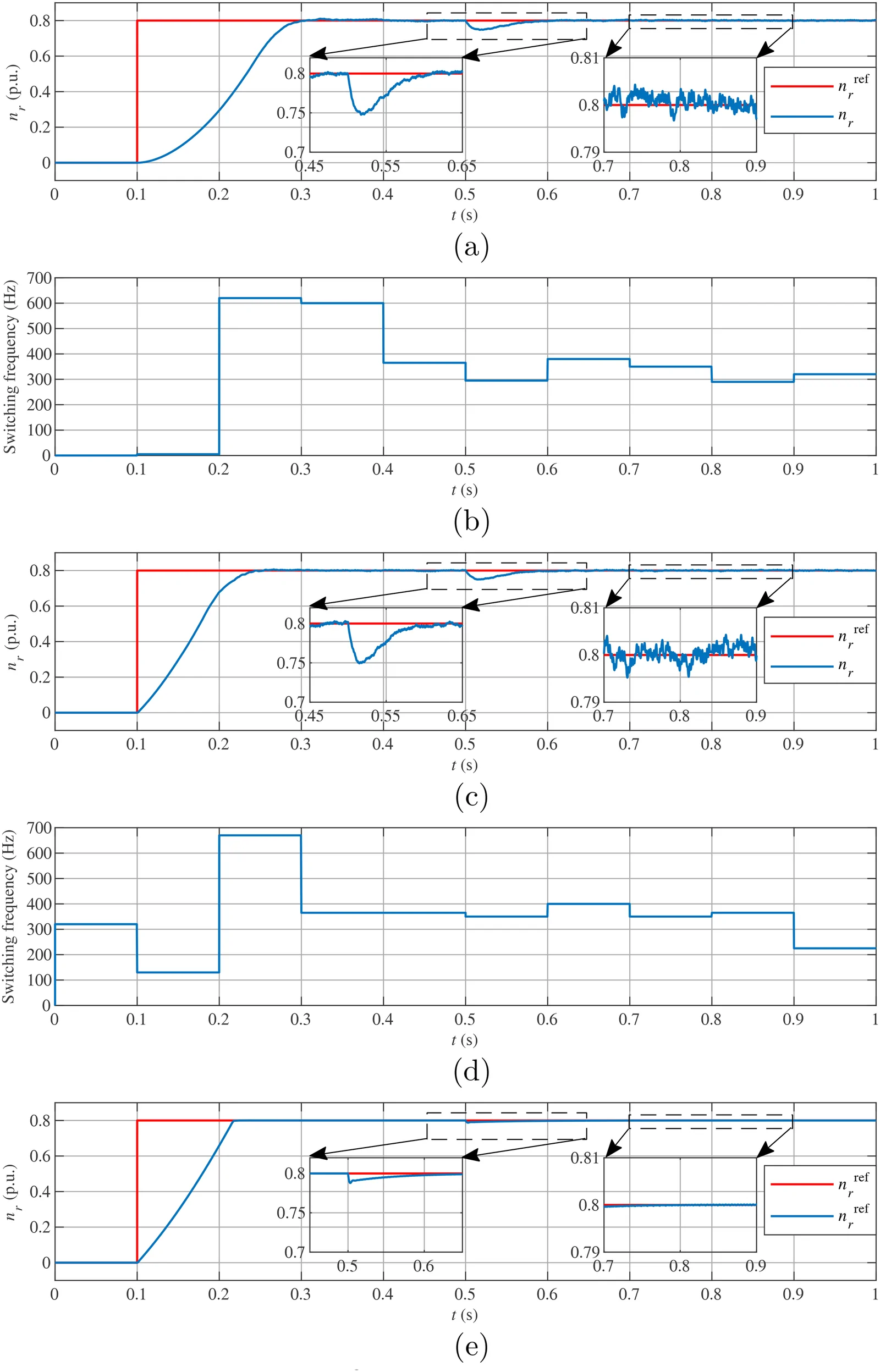
Simulation results of motor speed with zoomed-in views and equivalent switching frequency under 110 m long-cable PWM-driven system. (a) Speed for traditional MPCC. (b) Switching frequency for traditional MPCC. (c) Speed for improved MPCC. (d) Switching frequency for improved MPCC. (e) Speed for SVPWM.

**Fig 18:**
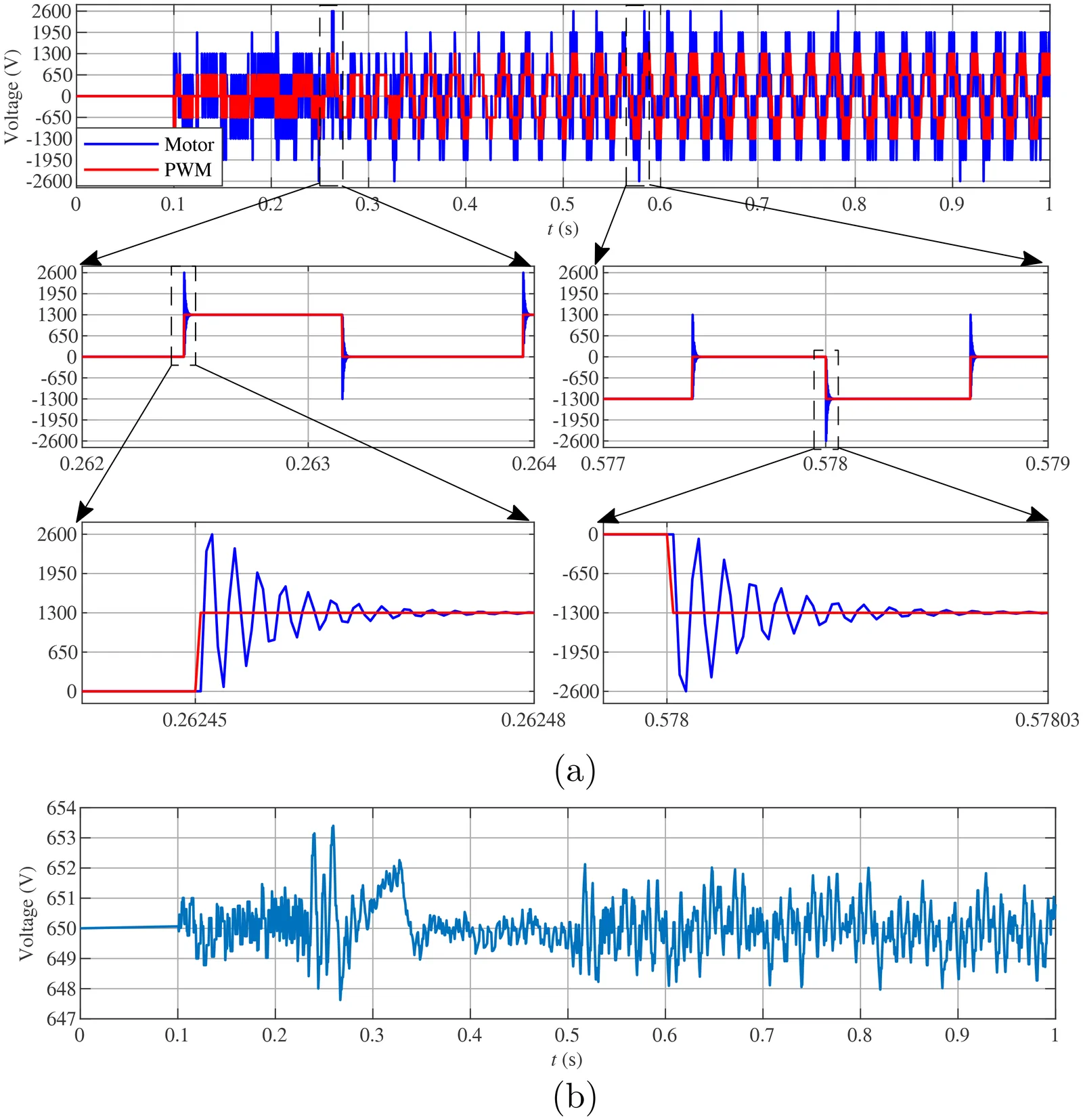
Simulation results of voltages in the traditional MPCC 110 m long-cable PWM-driven system. (a) Inverter and motor voltages with zoomed-in views. (b) Neutral-point voltage.

**Fig 19:**
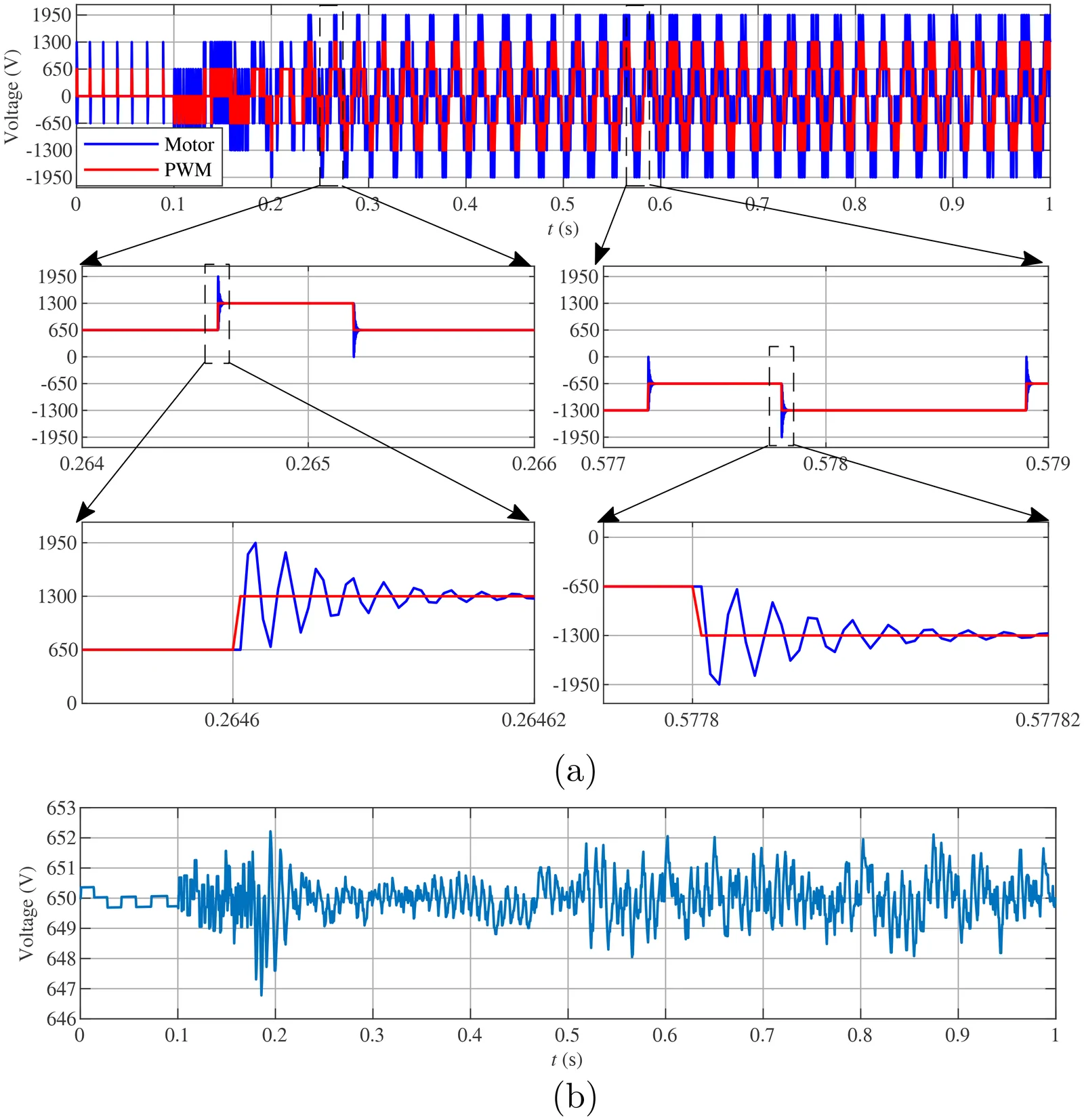
Simulation results of voltages in the improved MPCC 110 m long-cable PWM-driven system. (a) Inverter and motor voltages with zoomed-in views. (b) Neutral-point voltage.

**Fig 20:**
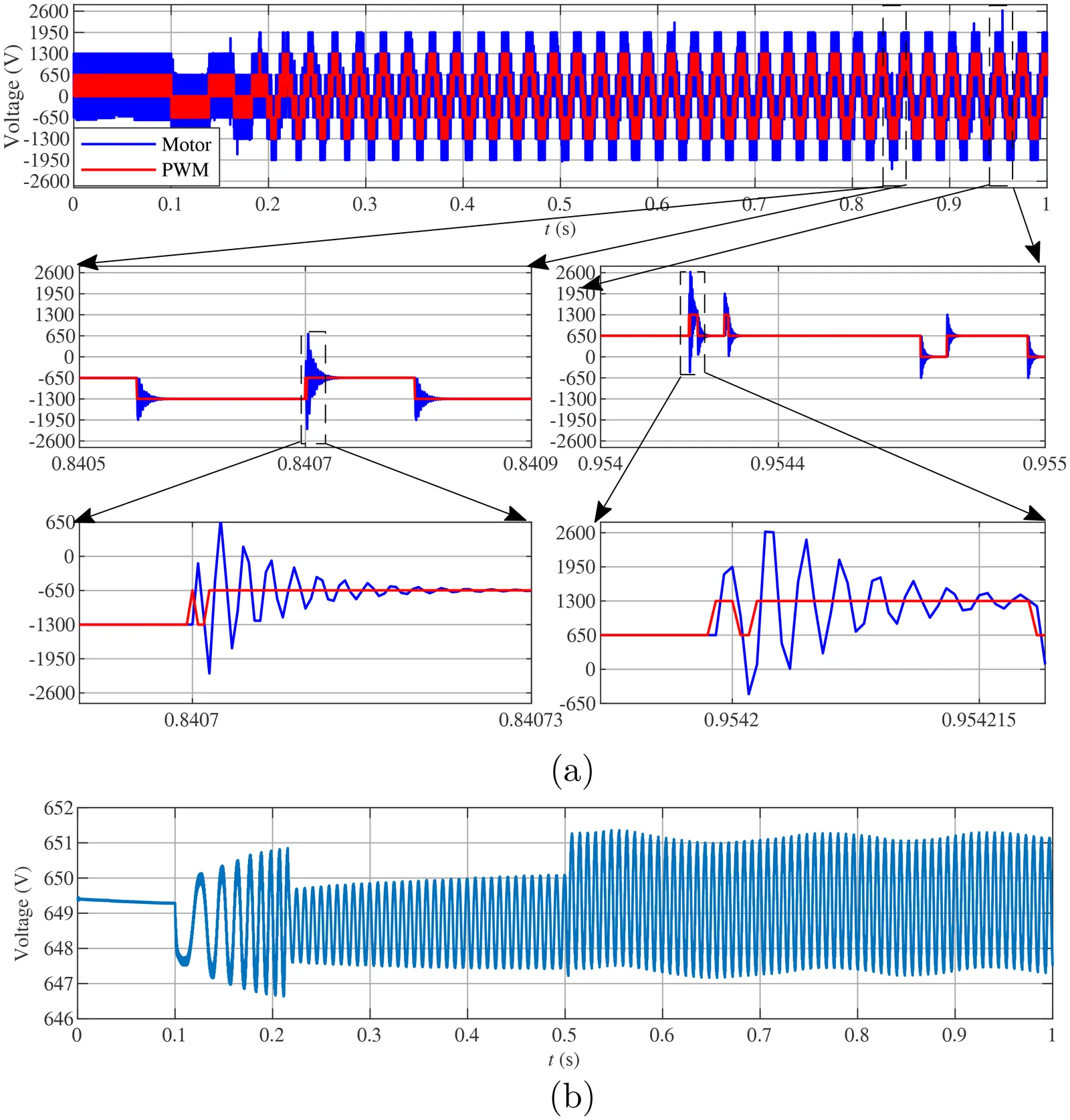
Simulation results of voltages in the 110 m long-cable SVPWM-driven system. (a) Inverter and motor voltages with zoomed-in views. (b) Neutral-point voltage.

**Fig 21:**
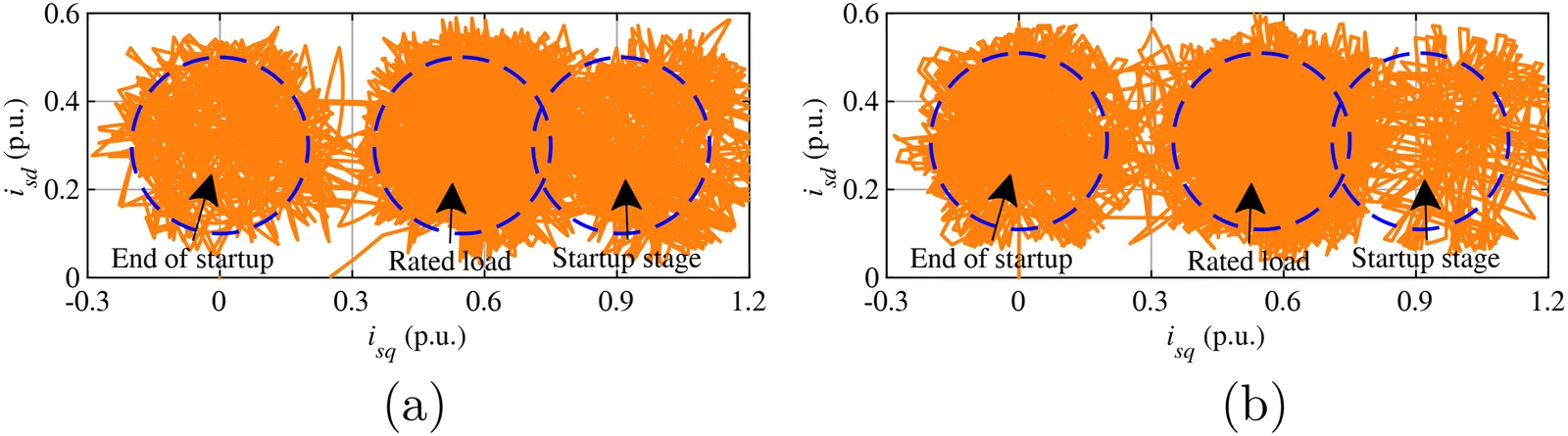
Simulation results of the stator d-q current trajectory with circular boundary predictive control in 110 m long-cable PWM-driven system. (a) Traditional MPCC. (b) Improved MPCC.

As shown in Figs 17(a), 17(c) and 17(e), the motor speed can track the reference of 0.8 p.u. under traditional MPCC, improved MPCC and SVPWM strategies. The speed tracking performance in simulation exhibits better trends: the maximum speed deviation of the two MPCC schemes is around 0.05 p.u., with steady-state deviation within 0.005 p.u. The SVPWM method shows preferable speed tracking performance, with a maximum deviation of approximately 0.01 p.u.

Figs 17(b) and 17(d) present the equivalent switching frequency under the three strategies. The traditional MPCC yields a maximum switching frequency of 620 Hz during startup (0.1–0.3 s), and fluctuates within 290–380 Hz with an average of 335.0 Hz under steady-state conditions (0.6–1.0 s). For the improved MPCC, the peak frequency is 670 Hz during startup, and maintains a range of 225–400 Hz with an average of 335.0 Hz at steady state. The SVPWM strategy operates at a fixed switching frequency of 2 kHz. Compared with the fixed 2 kHz switching frequency of SVPWM, both MPCC strategies achieve an average switching frequency of approximately 335 Hz, which accounts for only about 16.8% of that of SVPWM. This confirms the effectiveness of both MPCC schemes in significantly reducing switching losses, while the improved MPCC achieves comparable switching frequency performance to the traditional MPCC.

Figs 18–20 illustrate the NPC-TLI output voltages, motor terminal voltages and their zoomed-in views under the three control strategies. As depicted in Figs 18–20(a) and Table 4, the peak overvoltage at the motor terminal for traditional MPCC is 2598.6 V (2.89 p.u.), for SVPWM is 2619.8 V (2.91 p.u.), and for improved MPCC is 1950.1 V (2.17 p.u.). The improved MPCC strategy achieves a 24.9% reduction compared with traditional MPCC and 25.4% compared with SVPWM. This demonstrates that the improved MPCC effectively mitigates the overvoltage reflection effect at the motor terminal even without an RLC filter, confirming its inherent overvoltage suppression capability through active optimization of the voltage vector switching path. The overvoltage exceeds the insulation safety limit (>1836 V) under all three strategies at this cable length without filtering, the duration beyond limit voltage is 0.0154%, 0.0141% and 0.1173%, and thee duration beyond high-stress region (>1350 V) is 0.16%, 0.18% and 1.49%, respectively, indicating that an RLC filter remains necessary for full insulation safety compliance under more demanding conditions.

**Table 4:** Comparison of simulation result characteristics for traditional MPCC, improved MPCC and SVPWM strategies in 110 m cable system.

Key metric	Traditional MPCC	Improved MPCC	SVPWM
Peak voltage (V)	2598.6	1950.1	2619.8
Overvoltage ratio (relative to 900 V)	2.89	2.17	2.91
Safety voltage margin (V) (relative to 1836)	−762.6	−114.1	−783.8
Duration beyond limit voltage (>1836 V)	0.0154%	0.0141%	0.1173%
Duration beyond high‑stress region (>1350 V)	0.16%	0.18%	1.49%
Neutral-point voltage (V)	647.6–653.4 (mean 650.1)	646.8–652.2 (mean 650.0)	646.6–651.4 (mean 649.0)
Equivalent switching frequency (Hz) (steady-state: 0.6–1 s)	290.0–380.0 (mean 335.0)	225.0–400.0 (mean 335.0)	2000

Figs 18–20(b) and Table 4 present the neutral-point voltage waveforms in simulation. The traditional MPCC maintains the neutral-point voltage within 647.6–653.4 V (mean 650.1 V), the improved MPCC within 646.8–652.2 V (mean 650.0 V), and SVPWM within 646.6–651.4 V (mean 649.0 V). The improved MPCC exhibits the smallest neutral-point voltage fluctuation and maintains the voltage closest to the ideal reference of 650 V.

Fig 21 shows the stator current trajectories in the $i_{d}$–$i_{q}$ plane under traditional and improved MPCC strategies. Both methods can limit the current error within a certain range, with the improved MPCC showing slightly better current regulation performance.

The coupling effects between the RLC filter and cable length are further summarized from Tables 3 and 4 as follows. At 1 km with the RLC filter, the improved MPCC achieves the lowest peak voltage (1090.4 V, 1.21 p.u.), compared with 1135.4 V (1.26 p.u.) for traditional MPCC and 1216.6 V (1.35 p.u.) for SVPWM. The safety margins are 745.6 V, 700.6 V, and 619.4 V, respectively. The duration beyond the limit voltage and the high-stress region (>1350 V) are all 0% under all three strategies. The equivalent switching frequency of the improved MPCC is 760.0 Hz (mean), reduced by 11.9% compared with traditional MPCC (862.8 Hz) and by 62.0% compared with SVPWM (2000 Hz fixed).

At 110 m without the RLC filter, the improved MPCC achieves the lowest peak voltage (1950.1 V, 2.17 p.u.), compared with 2598.6 V (2.89 p.u.) for traditional MPCC and 2619.8 V (2.91 p.u.) for SVPWM. The safety margins are −114.1 V, −762.6 V, and −783.8 V, respectively. The duration beyond the limit voltage (>1836 V) is 0.0141% for the improved MPCC, compared with 0.0154% for traditional MPCC and 0.1173% for SVPWM. The high-stress region (>1350 V) duration is 0.18% for the improved MPCC, compared with 0.16% for traditional MPCC and 1.49% for SVPWM. The equivalent switching frequency of both MPCC strategies is 335.0 Hz, representing a reduction of 83.3% compared with SVPWM (2000 Hz fixed).

The RLC filter and the proposed MPCC exhibit a synergistic coupling. At 1 km with the RLC filter, the improved MPCC reduces the overvoltage peak by 4.0% compared with traditional MPCC and by 10.4% compared with SVPWM. At 110 m without the RLC filter, the improvements are more pronounced: 24.9% and 25.4%, respectively. This indicates that the improved MPCC provides stronger overvoltage suppression when the cable reflection effect is more severe. When the RLC filter is present, the absolute overvoltage levels are lower, but the relative improvement of the improved MPCC remains consistent, confirming that the strategy synergistically enhances the filter’s effectiveness. The results also confirm that the RLC filter remains an essential component for ensuring insulation safety under long-cable conditions, as the overvoltage exceeds the safety limit at 110 m without the RLC filter under all three strategies.

### Experimental verification of 110 m cable system without an RLC filter

The experimental test rig is configured as follows: the sampling and control core consists of a DSP TMS320F28379D and an FPGA EP4CE22E22C8N, responsible for signal acquisition, algorithm implementation, and PWM signal generation. The main power devices are Infineon FF600R17ME4 IGBT modules (1700 V/600 A), paired with Power Integrations 2SC0435T2F1-17 driver cores. The DC-link employs HCGFA 1500 μF/450 V electrolytic capacitors, each paralleled with a 7.2 kΩ/20 W balancing resistor. A pre-charge circuit using a 20 Ω/100 W aluminum-housed resistor and a bypass contactor is also incorporated. Voltage measurements are performed by TV30GB transformers (3000 V, ±1% accuracy), while current measurements are carried out by LT 1005-S Hall-effect current sensors. The ESP motor employed in the tests is the YQY114J model, whose ratings are summarized in Table 2.

It should be emphasized that, due to limitations of the experimental setup, the actual cable length used in the experiments is 110 m, which far exceeds the maximum allowable cable length (approximately 50 m) for conventional PWM-driven systems, and the test was conducted without an RLC filter. Although the cable length is different from the 1 km adopted in simulation, the primary purpose of the experiments is to validate the basic functionality of the improved MPCC strategy, i.e., its capability to suppress motor terminal overvoltage and reduce equivalent switching frequency in PWM-driven systems with long or limited cable lengths.

The corresponding experimental test results are illustrated in Figs 22–26, respectively, where the reference values, SVPWM switching and sampling frequencies adopted in the experimental test are consistent with those in the simulation.

**Fig 22:**
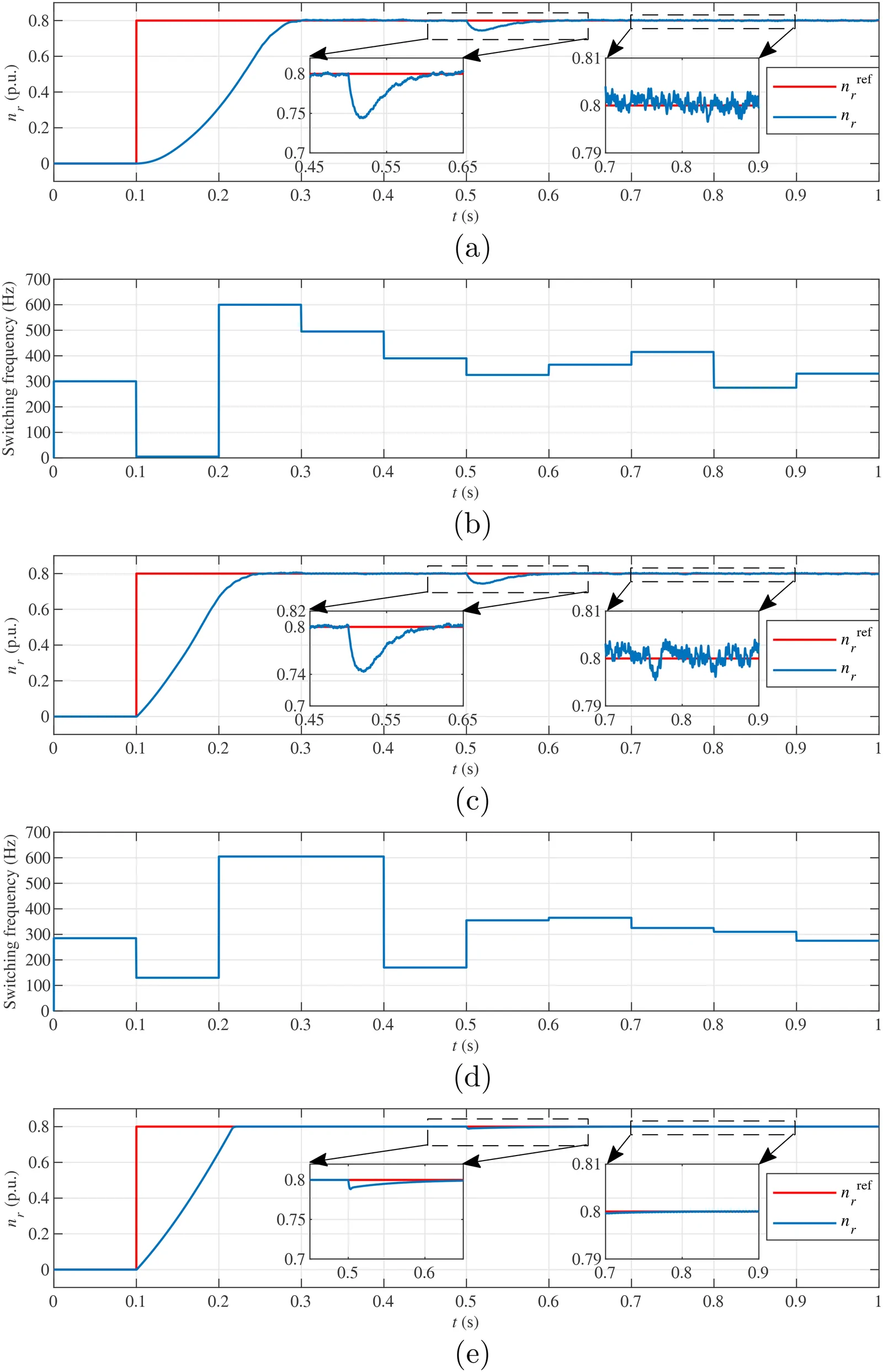
Experimental results of motor speed with zoomed-in views and equivalent switching frequency under 110 m long-cable PWM-driven system. (a) Speed for traditional MPCC. (b) Switching frequency for traditional MPCC. (c) Speed for improved MPCC. (d) Switching frequency for improved MPCC. (e) Speed for SVPWM.

**Fig 23:**
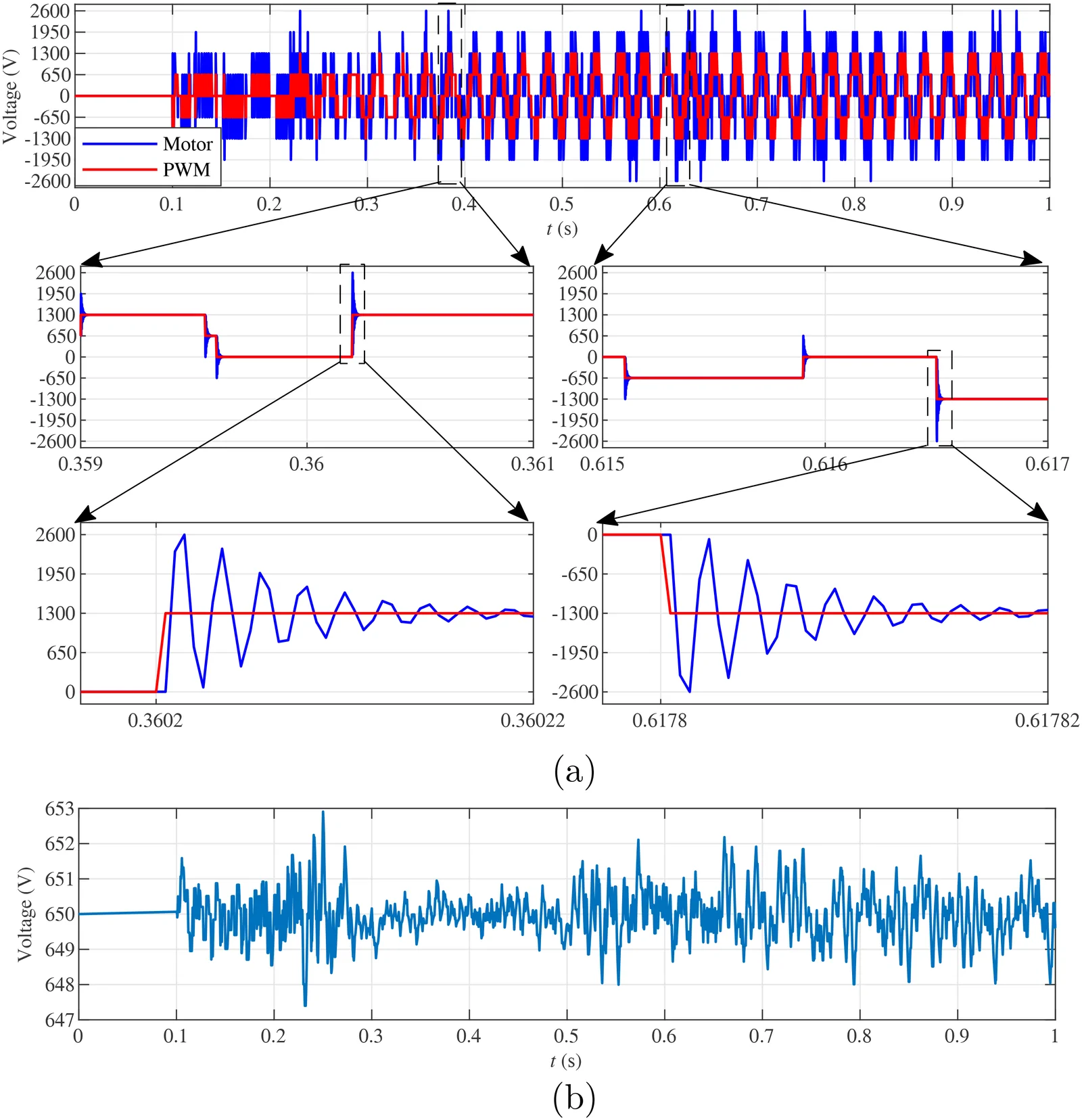
Experimental results of voltages in the traditional MPCC 110 m long-cable PWM-driven system. (a) Inverter and motor voltages with zoomed-in views. (b) Neutral-point voltage.

**Fig 24:**
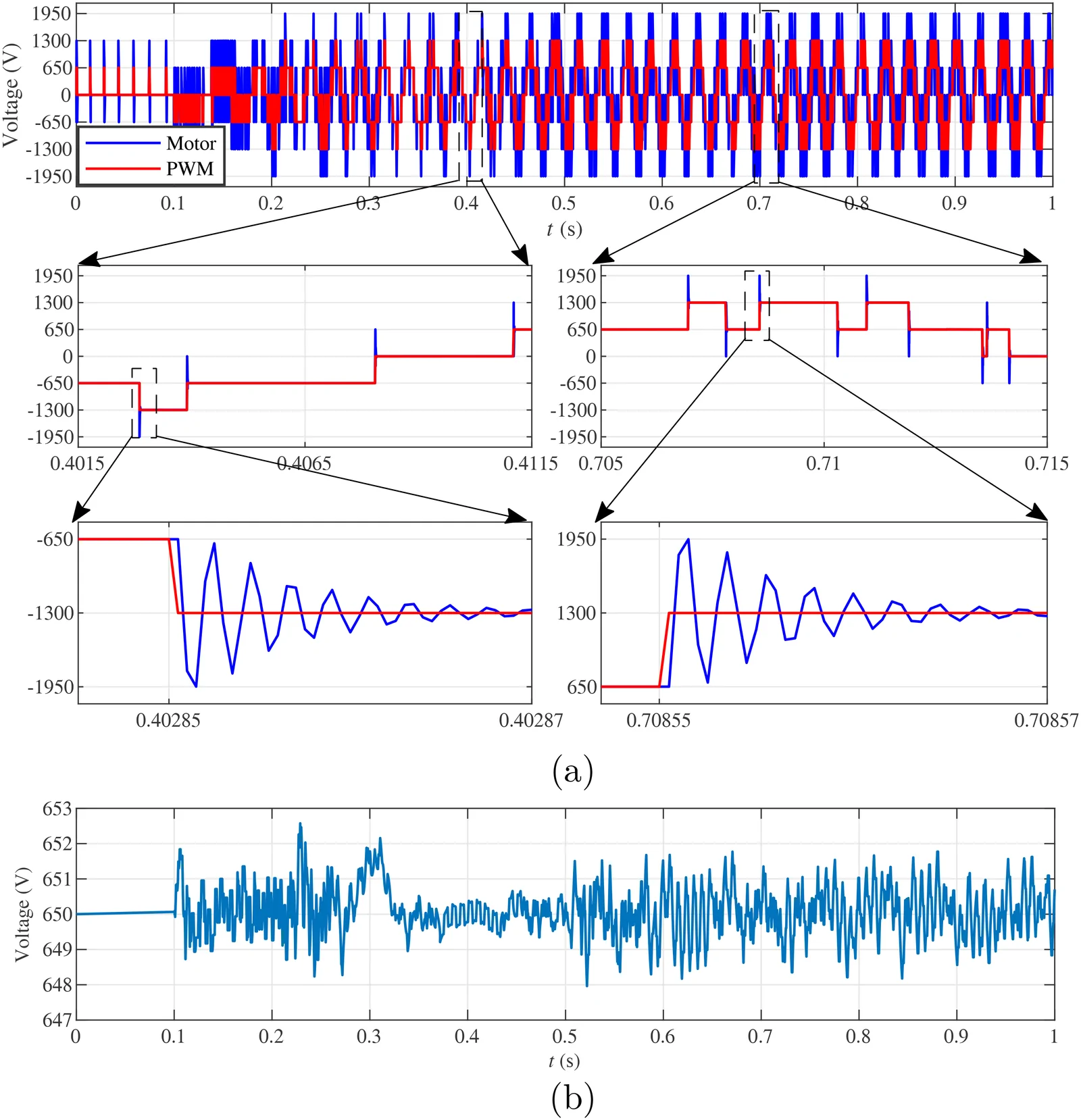
Experimental results of voltages in the improved MPCC 110 m long-cable PWM-driven system. (a) Inverter and motor voltages with zoomed-in views. (b) Neutral-point voltage.

**Fig 25:**
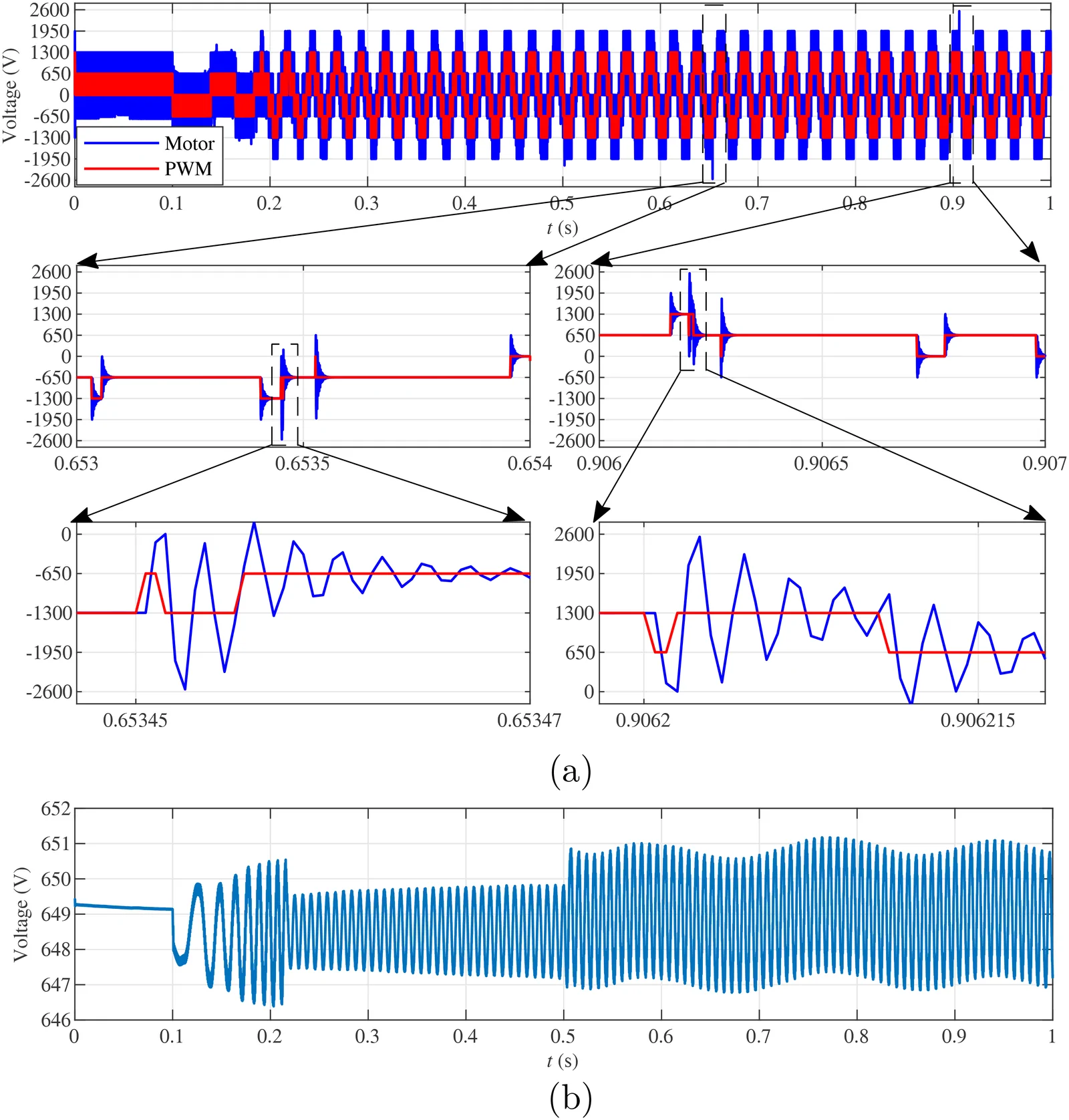
Experimental results of voltages in the 110 m long-cable SVPWM-driven system. (a) Inverter and motor voltages with zoomed-in views. (b) Neutral-point voltage.

**Fig 26:**
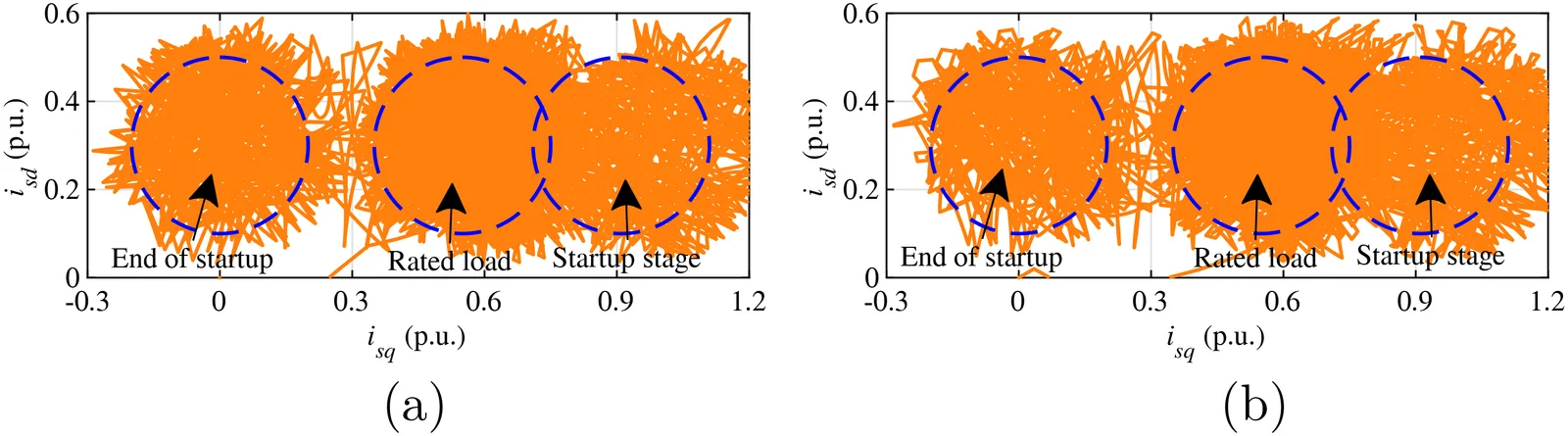
Experimental of the stator d-q current trajectory with circular boundary predictive control in 110 m long-cable PWM-driven system. (a) Traditional MPCC. (b) Improved MPCC.

As depicted in Fig 22(a), 22(c) and 22(e), the motor speed can well track the reference of 0.8 p.u. under traditional MPCC, improved MPCC and SVPWM strategies. The maximum speed deviation of the two MPCC schemes is around 0.05 p.u., with steady-state deviation ranging from 0.005 p.u. By comparison, the SVPWM method exhibits preferable speed tracking performance, whose maximum speed deviation is only 0.02 p.u. In Fig 22(b) and 22(d), the traditional MPCC yields a maximum equivalent switching frequency of 600 Hz during the startup 0.1–0.3 s. In steady-state operation from 0.6–1 s, its switching frequency fluctuates within 275.0–415.0 Hz, with an average value of 346.2 Hz. For the improved MPCC, the peak frequency is 600 Hz in the same startup interval, and maintains a range of 275.0–365.0 Hz with an average of 318.8 Hz under steady-state conditions. Obviously, the improved MPCC presents the minimum average equivalent switching frequency in steady state, its value accounts for roughly 92% of that for traditional MPCC and 16% for SVPWM strategy (2 kHz), which will effectively reduce the switching losses.

Figs 23–25 illustrate the output voltages of NPC-TLI and motor terminal as well as their zoomed-in views, and the neutral-point voltages under three control strategies. As observed in Figs 23–25(a), unlike the 1 km long-cable condition, the rise time of the PWM pulses and the cable propagation delay fall into a specific matching interval at a cable length of 110 m. After the inverter output PWM voltage is transmitted through the 110 m cable to the motor, overvoltage spikes appear at the motor terminal under all three control strategies. For the traditional MPCC, the peak overvoltage at the motor terminal is 2598.7 V, reaching 2.89 times the rated voltage (900 V); for the SVPWM, the peak overvoltage is 2561.1 V, 2.85 times the rated voltage; however, for the improved MPCC, the peak overvoltage is only 1950.1 V, 2.17 times the rated voltage. These quantitative data reveal that the improved MPCC strategy achieves the lowest peak overvoltage (1950.1 V), representing a 25.0% reduction compared with traditional MPCC and 23.9% for SVPWM. Under all three strategies, the overvoltage exceeds the insulation safety limit (1836 V), indicating a risk of motor insulation breakdown, with all safety margins being negative (as denoted in Table 5). Where, the improved MPCC strategy exhibits the largest safety margin (−114.1 V), with an exposure duration beyond the limit voltage (>1836 V) of 0.0132%, a high-stress zone (>1350 V) ratio of 0.17%, and thus the lowest insulation risk. However; the traditional MPCC strategy shows the smallest safety margin (−762.7 V), its exposure duration ratio beyond the limit and high-stress zone (>1350 V) ratio are is 0.0173%, 0.17%, respectively. Furthermore, the SVPWM strategy has a moderate safety margin (−725.1 V), the highest exposure duration ratio beyond the limit (0.1181%), and the highest high-stress zone ratio (1.48%).

**Table 5:** Comparison of experimental result characteristics for traditional MPCC, improved MPCC and SVPWM strategies in 110 m cable system.

Key metric	Traditional MPCC	Improved MPCC	SVPWM
Peak voltage (V)	2598.7	1950.1	2561.1
Overvoltage ratio (relative to 900 V)	2.89	2.17	2.85
Safety voltage margin (V) (relative to 1836)	−762.7	−114.1	−725.1
Duration beyond limit voltage (>1836 V)	0.0173%	0.0132%	0.1181%
Duration beyond high‑stress region (>1350 V)	0.17%	0.17%	1.48%
Neutral-point voltage (V)	647.4–652.9 (mean 650.0)	647.9–652.6 (mean 650.0)	646.4–651.2 (mean 648.8)
Equivalent switching frequency (Hz) (steady-state: 0.6–1 s)	275.0–415.0 (mean 346.2)	275.0–365.0 (mean 318.8)	2000

Figs 23–25(b) present the neutral-point voltage waveforms under three different control strategies: for the traditional MPCC, the neutral-point voltage fluctuates within 647.4–652.9 V with an average value of 650.0 V; for the improved MPCC strategy, the neutral-point voltage varies from 647.9–652.6 V and has an average value of 650.0 V; in terms of the SVPWM control strategy, its neutral-point voltage ranges between 646.4–651.2 V, with an average value of 648.8 V. As a result, the improved MPCC features smaller neutral-point voltage fluctuations and keeps the operating voltage closer to the reference value of 650 V.

Fig 26 shows the stator current trajectories in the $i_{d}$-$i_{q}$ plane under traditional and improved MPCC strategies, indicating that both methods can limit the current error within a certain range.

A comparison of Tables 3–5 shows that the proposed improved MPCC consistently outperforms both the traditional MPCC and SVPWM strategies across all configurations. The advantages of the improved MPCC strategy in overvoltage suppression and equivalent switching frequency reduction become more pronounced as the cable length increases, confirming its scalability to more demanding long-cable conditions.

It should be noted, however, that even with the improved MPCC strategy, the motor terminal overvoltage at 110 m without an RLC filter (1950.1 V) still exceeds the insulation safety limit of 1836 V, resulting in a negative safety margin (−114.1 V). This indicates that software optimization alone cannot completely eliminate the overvoltage risk in long-cable PWM-driven systems; the RLC filter remains an essential component for ensuring long-term insulation reliability. Nevertheless, the improved MPCC significantly reduces the overvoltage peak and its duration compared with traditional MPCC and SVPWM, thereby alleviating the filtering burden and extending the operational margin when used in conjunction with the RLC filter.

Based on these findings, the improved MPCC strategy not only enhances overvoltage suppression and optimizes switching frequency but also maintains neutral-point voltage balance, demonstrating its clear technical superiority and practical engineering value for ESP drive systems under varying cable lengths.

## Conclusions

This paper has systematically investigated the overvoltage suppression problem in long-cable PWM-driven induction motor systems for electrical submersible pump (ESP) applications and proposed an improved model predictive current control (MPCC) strategy. The distributed parameter effects of long cables cause impedance mismatch between the inverter and the motor, leading to voltage reflection and transient overvoltages up to 2–3 times the DC bus voltage.

By actively optimizing the voltage vector switching path, the proposed improved MPCC strategy reduces the overvoltage peak ratio from 2.85–2.91 to 2.17 at 110 m cable length, and from 1.26–1.35 to 1.21 at 1 km cable length, representing reductions of 24.9–25.4% and 4.0–10.4%, respectively, compared with the traditional MPCC and SVPWM strategies. The equivalent switching frequency of the improved MPCC is reduced to 92.1% and 88.1% of that of the traditional MPCC at 110 m and 1 km, respectively, while the neutral-point voltage fluctuation is maintained within a narrow range around 650 V.

As the cable length increases, the advantages of the improved MPCC strategy become increasingly significant. The strategy is applicable over a range of cable length variations without requiring hardware filter redesign. In summary, the improved MPCC strategy, in conjunction with an RLC filter, provides an effective solution for long-cable PWM-driven ESP systems, significantly suppressing overvoltage, reducing switching losses, and enhancing system reliability for deep-well oil extraction applications.

It should be noted that the present study focuses on the development and validation of the proposed strategy under nominal motor parameters. A comprehensive parameter sensitivity analysis and online identification scheme for high-temperature ESP applications are currently under investigation and will be reported in future work.
